# Revealing Glycosylation Patterns in *In Vitro*-Produced Mucus Exposed to Pasteurized Mucus-Associated Intestinal Microbes by MALDI-TOF-MS and PGC-LC-MS/MS

**DOI:** 10.1021/acs.jafc.4c01401

**Published:** 2024-06-27

**Authors:** Carol de Ram, Benthe van der Lugt, Janneke Elzinga, Sharon Geerlings, Wilma T. Steegenga, Clara Belzer, Henk A. Schols

**Affiliations:** † Laboratory of Food Chemistry, Wageningen University & Research, Bornse Weilanden 9, 6708 WG Wageningen, The Netherlands; ‡ Laboratory of Microbiology, Wageningen University & Research, Stippeneng 4, 6708 WE Wageningen, The Netherlands; § Human Nutrition and Health, Wageningen University & Research, Stippeneng 4, 6708 WE Wageningen, The Netherlands

**Keywords:** mucin, mucus, glycosylation, intestinal microbiota, *Akkermansia muciniphila*, mucosal health

## Abstract

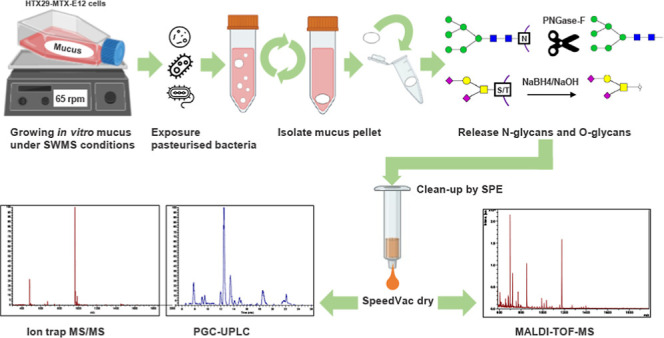

The human intestinal mucus layer protects against pathogenic microorganisms and harmful substances, whereas it also provides an important colonization niche for mutualistic microbes. The main functional components of mucus are heavily glycosylated proteins, called mucins. Mucins can be cleaved and utilized by intestinal microbes. The mechanisms between intestinal microbes and the regulation of mucin glycosylation are still poorly understood. In this study, *in vitro* mucus was produced by HT29-MTX-E12 cells under Semi-Wet interface with Mechanical Stimulation. Cells were exposed to pasteurized nonpathogenic bacteria *Akkermansia muciniphila*, *Ruminococcus gnavus*, and *Bacteroides fragilis* to evaluate influence on glycosylation patterns. Following an optimized protocol, O- and N-glycans were efficiently and reproducibly released, identified, and semiquantified using MALDI-TOF-MS and PGC-LC-MS/MS. Exposure of cells to bacteria demonstrated increased diversity of sialylated O-glycans and increased abundance of high mannose N-glycans in *in vitro* produced mucus. Furthermore, changes in glycan ratios were observed. It is speculated that bacterial components interact with the enzymatic processes in glycan production and that pasteurized bacteria influence glycosyltransferases or genes involved. These results highlight the influence of pasteurized bacteria on glycosylation patterns, stress the intrinsic relationship between glycosylation and microbiota, and show the potential of using *in vitro* produced mucus to study glycosylation behavior.

## 1 Introduction

**Figure 1:**
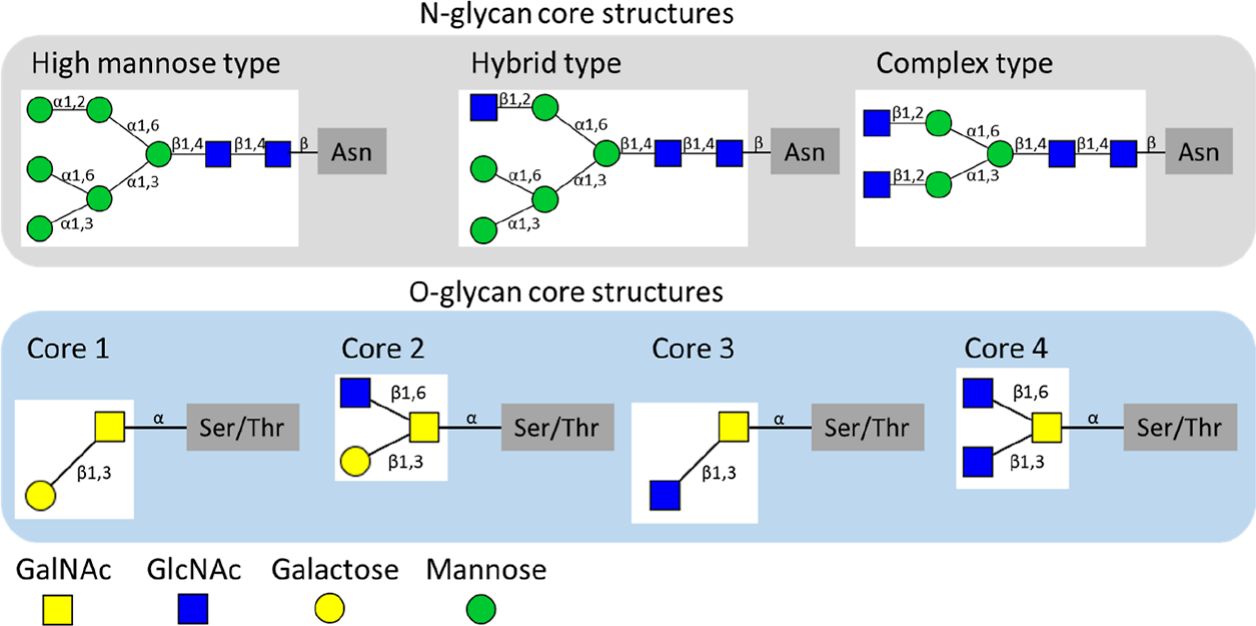
Visualization of the three core N-glycan structures (high mannose, hybrid, complex) and the four most common O-glycan core structures (1–4) occurring in mucins.

In recent years, the intestinal mucus barrier has gained increasing attention for its vital role in sustaining intestinal health.^1^ Mucus, produced by specialized goblet cells, is a complex viscous secretion that covers the intestinal epithelial surface and thereby fulfils a crucial function as a physical protective barrier.^2,3^ Mucus is present, among others, throughout the human gastrointestinal tract (GIT) and it can vary greatly in structure and thickness.^4^ The main functional and structural components of mucus are mucins, large glycosylated proteins.^5^ There are two classified groups of mucins: transmembrane and secreted mucins. Transmembrane mucins are primarily involved in cellular adhesion, while the secreted mucins are mostly responsible for the viscoelasticity of the mucus layer.^6^ The secreted gel forming mucin type 2 (MUC2) is the most abundant component of colonic mucus.^7^ In this study, the focus is on colonic mucins. The mucin polypeptide backbone is structured as a core domain of repetitive tandem units (PTS domains) of proline (Pro), threonine (Thr), and serine (Ser).^8^ In between and at the terminus of the PTS domains, cysteine-rich regions are located.^5,9^ Mucins are modified by N- and O-glycosylation, both essential for the mucin properties.^10^ N-glycosylation is especially important during mucin peptide processing and is essential for proper folding and dimerization of the MUC2 mucin.^10,11^ N-glycans consist of three core structures, as shown in Figure 1, which can be further extended by enzymes adding sugars to the glycan core, elongating branching residues by sugar addition, and “decoration” of the elongated branches.^10^ O-glycosylation is mainly responsible for shaping and maintaining the mucin 3-D structure.^12^ Mucin O-glycan structures consist mostly of the four common subtypes core 1–4 as shown in Figure 1 (and Supporting Information Figure S1).^13^ These O-glycan core structures are based on a GalNAc residue extended with galactose and/or GlcNAc and decorated by fucosyl, sialyl, and sulfate substituents. These decorations protect the mucin O-glycan from being easily degraded and affect the physicochemical properties of the mucin. The abundance and heterogeneity of fucosylation in the human GIT was shown lowest for colonic mucus, whereas sialylation was highest for colonic mucus compared to mucus from other parts of the human GIT. Mucus from different origins may vary in the dominant type of O-glycan core structures present as well.^4,14^ Stomach mucus, for example, contains primarily core 1 and core 2 based structures, whereas colonic mucus contains predominantly core 3 and core 4-based structures.^4^

Mucus and mucin glycosylation is influenced by various factors of which microbial interaction is especially important.^3,8,15^ Pathogens, as well as, the intestinal microbiota can use mucin substituents as attachment site.^16^ Furthermore, the intestinal microbiota can use mucins as an important nutrient source^17−19^ and they can utilize monosaccharides released from the glycans to produce short-chain fatty acids to be used by other microorganisms (cross-feeding) as well as by the host itself.^20,21^ Thus, there is an intrinsic dynamic relationship between the mucin glycans and the composition of the intestinal microbiota.^22−25^ For example, the presence of *Akkermansia muciniphila* is associated with increased mucus thickness and increased number of goblet cells.^1,26^*A. muciniphila*, *Ruminococcus gnavus*, and *Bacteroides fragilis* are, among others, capable of using and degrading mucin glycans.^27−30^*A. muciniphila* and *B. fragilis* are associated with intestinal health benefits such as prevention of inflammation.^31,32^ Furthermore, it has been shown that even pasteurized bacteria can still practice beneficial effects. Their outer membrane components, e.g., pili, fimbriae, lipopolysaccharides (LPS), and extracellular vesicles, can signal host responses and have therapeutical potential.^33,34^ Additionally, in terms of application, pasteurized bacteria are safer than their living counterparts.^31^ Several studies have presented preliminary data that supports the hypothesis that dysbiosis in intestinal microbiota and certain variation in mucin O-glycosylation patterns are associated with diseases such as irritable bowel syndrome and colorectal cancer.^3,23^ This all correlates with the hypothesis that mucin glycosylation could be a powerful indicator and a valuable target to improve intestinal health.^24,35^ However, the exact interaction mechanisms between intestinal microbiota and mucin glycosylation are still poorly understood. Two important challenges in mucin analysis are the complexity of the mucus structure and the difficulty in quantitative and qualitative analyses of mucin glycans. This is essential to understand and modify the interaction between the intestinal microbiota and mucins in the context of overall gut health.^23,36,37^

The aim of this study was to characterize and semiquantify glycans present in mucus and evaluate the influence of intestinal microbes on the glycosylation patterns. For this purpose, the mucus-secreting colonic cancer cell line HT29-MTX-E12 was used. This cell line predominantly secretes gel-forming mucins MUC5AC and MUC5B as well as various transmembrane mucins.^20,38^ Growth of cell line HT29-MTX-E12 applying Semi-Wet interface with Mechanical Stimulation (SWMS) on Transwell membranes showed a more coherent mucus layer and increased overall mucin production.^38,39^ Increased production of MUC2 and decreased production of, among others, MUC5AC was observed.^20^ In the current study, HT29-MTX-E12 cells were grown in cell-culture flasks under adapted SWMS. Methods for the characterization and semiquantification of O- and N-linked glycans from *in vitro* produced mucus expressed by HT29-MTX-E12 cells grown in culture flasks under SWMS were modified, optimized, and validated.^20^ Furthermore, the effects of exposure to pasteurized nonpathogenic intestinal mucus-associated bacteria *A. muciniphila*, *R. gnavus*, and *B. fragilis* were studied in relation to the presence and abundance of characterized glycans. This research was performed using a fully optimized and dedicated approach utilizing *in vitro* produced mucus, release of O- and N-linked glycans, purification by solid phase extraction, and characterization and semiquantification using MALDI-TOF-MS and PGC-LC-MS/MS.

## 2 Materials and Methods

### 2.1 Chemicals

Water (H_2_O), acetonitrile (ACN), methanol (MeOH), acetic acid, and trifluoroacetic acid (TFA) ULC/MS-CC/SFC grade ≥99% were all obtained from Biosolve (Dieuze, France). 2,5-Dihydroxybenzoic acid (DHB) ≥ 99.5% was obtained from Bruker (Bremen, Germany). Maltodextrin [degree of polymerization (DP) 1–20]from potato starch ≥99% pure (glucose) was obtained from Avebe (Veendam, The Netherlands). Fetuin from fetal bovine serum ≥99.7%, 1,4-α-D-maltopentaose 99% (DP5), 1,4-α-D-maltoheptaose 99% (DP7), mucin type III from porcine stomach partially purified powder, ammonium bicarbonate (NH_4_HCO_3_) BioUltra ≥98%, sodium borohydride (NaBH_4_) caplets 98%, sodium chloride (NaCl) ≥ 98%, sodium hydroxide (NaOH) ≥ 98% pellets, sodium dodecyl sulfate (SDS) ≥ 97%, IGEPAL CA-630 molecular biology grade, PNGase F enzyme from *Elizabethkingia meningoseptica* proteomics grade, and Supelco Supelclean Envi-Carb solid phase extraction (SPE) tubes 250 mg 3 mL were all obtained from Sigma-Aldrich (Darmstadt, Germany). Sep-Pak Vac 6 cm^3^ 500 mg 6 mL C18 SPE cartridges were obtained from Waters (Eschborn, Germany). BGB 0.2 mL PP short thread vials 32 × 11.6 mm and BGB ND9 short thread screw caps with slitted septa silicone/PTFE were obtained from Thermo Scientific (San Jose, CA, USA). 1.5 and 2.0 mL Eppendorf Safe-Lock microcentrifuge tubes were obtained from VWR (Boxmeer, The Netherlands). For incubation of samples at set temperature, a 1.5 and 2.0 mL Thermomixer comfort was used (Eppendorf, Nijmegen). Mucus supernatant samples were centrifuged at 4500*g*, and all other centrifugation steps were performed at 14,000*g*. A Savant centrifugal evaporator from Thermo Scientific was used for drying the samples. For SPE, a Vacuum manifold from Waters (Massachusetts, USA) and a vacuum gas pump from VWR were used.

### 2.2 Bacterial Culture of *A. muciniphila*, *R. gnavus*, and *B. fragilis*

*A. muciniphila* (ATCC BAA-835) was cultivated using minimal medium supplemented with threonine and a mixture of GlcNAc and glucose. The medium consisted of 0.5 mg/L resazurin, 0.4 g/L KH_2_PO_4_, 0.669 g/L Na_2_HPO_4_ + 2H_2_O, 0.3 g/L NH_4_Cl, 0.3 g/L NaCl, 0.1 g/L MgCl_2_*6H_2_O, 6 g/L l-threonine, trace elements in acid (1 mg/L of a solution containing 50 mM HCl, 1 mM H_3_BO_3_, and 0.5 mM CuCl_2_·2H_2_O) and trace elements in alkaline (1 mg/L of a solution containing 10 mM NaOH, 0.1 mM Na_2_SeO_3_, 0.1 mM Na_2_WO_4_, and 0.1 mM Na_2_MoO_4_). The medium was autoclaved and supplemented with 1% of filter sterilized vitamin solution (11 g/L CaCl_2_, 20 mg of biotin, 200 mg of nicotinamide, 100 mg of *p*-aminobenzoic acid, 200 mg of vitamin B1, 100 mg of panthothenic acid, 500 mg of pyridoxamine, 100 mg of vitamin B12, and 100 mg of riboflavin), 25 mM GlcNAc, 25 mM glucose, and 10 g/L l-cysteine-HCl.^30^

*B. fragilis* (ATCC 25285) and *R. gnavus* (ATCC 29149) were cultivated on brain heart infusion-supplemented (BHIS) medium. The medium contained 37 g/L brain heart infusion broth, 5 g/L yeast extract, 1 mg/L resazurin, 0.5 g/L l-cysteine-HCl, a 10 mL/L hemin solution, and 0.2 mL/L vitamin K1. The hemin solution was prepared by addition of 50 mg of hemin and 1 mL of NaOH to 100 mL of distilled water. Vitamin K1 solution was prepared by addition of 0.15 mg of vitamin K1 to 30 mL of 95% EtOH.

All strains were cultivated under anaerobic conditions in serum bottles. These bottles had a headspace of mixed gas consisting of 80:20 CO_2_/N_2_. The different media were inoculated with 1% v/v glycerol stock containing one of the three bacteria. The inoculated bottles were incubated at 37 °C for 40 h without shaking. After the incubation step, the supernatant was separated by centrifuging the cultures repeatedly at 10,000*g* for 20 min at 4 °C until the supernatant was a clear solution. The bacteria were suspended in PBS at an OD value similar to 10^9^ cfu/mL of live equivalent. The bacteria were then pasteurized at 70 °C for 30 min. Bacteria solutions were then diluted 10× and 100× in PBS corresponding to an OD equal to a solution of 10^8^ and 10^7^ cfu/mL microbial cells respectively and stored at −20 °C until use. Colony-forming units were based on the OD_600_ values. For *A. muciniphila* OD_600_ = 3.6 × 10^8^ and for *R. gnavus* and *B. fragilis* OD_600_ = 2.4 × 10^9^. The effectivity of pasteurization was tested by plating samples on BHI/mucin (*A. muciniphila*) or BHIS (*R. gnavus* and *B. fragilis*) agar in duplicate and incubating these plates under anaerobic conditions at 37 °C.^20^

### 2.3 Human Cell Culture and *In Vitro* Mucus Collection

HT29-MTX-E12 cells (ECACC) were obtained from Sigma-Aldrich. Cells were cultured in Dulbecco’s modified Eagle medium with 4.5 g/L glucose, 110 mg/L sodium pyruvate, and 584 mg/L l-glutamine (Corning, NY, USA) supplemented with 10% Fetal bovine serum and 1% penicillin/streptomycin. When cells reached 80–90% confluency, they were counted and seeded. Passage numbers between 3 and 21 were used for HT29-MTX-E12 cells. Cells were seeded in 75 cm^2^ flasks at a density of 5 × 10^6^ cells/flask in a volume of 12–15 mL. One day after seeding (day 1), media of all flasks was refreshed and an adapted version of SWMS was applied.^20^ The flasks were placed on a CO_2_-resistant shaker (Thermo Scientific) at 65 rpm. Medium was refreshed every Monday, Wednesday, and Friday. After 14 days in SWMS, the collection of produced mucus was started. First, the medium containing mucus was removed after thorough resuspending followed by the addition of 5 mL of fresh medium to the cells. This was performed every 2 days in the morning. The procedure was repeated five times yielding 25 mL of collected medium. The described collection was repeated for four individual batches. Collected mucus-medium was stored at −20 °C.

### 2.4 *In Vitro* Mucus Production with Exposure to Pasteurized Bacteria

After 14 days in SWMS, cells were also exposed to three selected pasteurized bacteria species (*Akkermansia mucinphila*, *R. gnavus*, and *B. fragilis*) in three concentrations (10^8^, 10^7^, and 10^6^ cfu/mL; see also bacterial culture). First, the present medium was removed and collected in a 50 mL tube. Then, 5 mL of diluted bacteria stock was added. This was performed every 2 days in the morning. The cells were exposed five times with diluted bacteria stock producing in total 25 mL collected medium per condition. The described collection was repeated for three individual batches. The collected mucus medium was stored at −20 °C.

### 2.5 Dot-Blot Assay for Determination of MUC2 and MUC5AC in *In Vitro* Mucus

A dot-blot assay was performed in order to test for the presence of MUC2 and MUC5AC in *in vitro* mucus. This was performed according to Elzinga et al.^20^ Approximately 10 mg of freeze-dried *in vitro* mucus pellet was dissolved in 1 mL of PBS and centrifuged at 14,000*g* for 30 min, and the supernatant was used for the dilution series on the membrane.

### 2.6 Isolation of *In Vitro* Produced Mucin

In order to isolate the mucins from the medium containing mucus, samples were thawed and carefully homogenized by incanting the tubes multiple times. Then, 12 mL of medium was pipetted into a 15 mL tube which was then centrifuged for 60 min at 4500*g*. The supernatant was removed from the “slimy” pelleted material. Subsequently, 8 mL H_2_O was added to the pellet, and the tubes were vortexed and centrifuged for 15 min at 4500*g*. This H_2_O washing was performed three times in total. The obtained “slimy” pellets were used for O- and N-linked glycan release. This described mucin isolation protocol was compared with the mucin extraction protocol as described by Ringot-Destrez et al.^40^ Applying both procedures for isolation and extraction of mucins from *in vitro* mucus produced by HT29-MTX-E12 cells under SWMS revealed similar results. The additional extraction steps described by Ringot-Destrez did not reveal additional glycan levels and/or structures, and therefore, mucins were isolated as described in this paragraph.

### 2.7 O-Glycan Release Using Reductive β-Elimination

To release the O-glycans, 800 μL of 1 M NaBH_4_ in 0.05 M NaOH was added to each sample. The main function of NaBH_4_ was to prevent peeling reactions of the released O-glycans. Furthermore, it assists in lowering the complexity of analysis, as no reducing end α/β-anomers are present after reduction. The samples were homogenized by pipetting up and down and subsequently 1 μL of 1 mg/mL DP5 internal standard was added. Then, the sample liquid was equally divided over 2 × 2 mL tubes and incubated overnight (18–22 h) at 45 °C at 300 rpm in a Thermomixer (Thermo Scientific). The following day, the samples were brought to neutral pH using glacial acetic acid, and the split samples were added back together. To each sample 250 μL H_2_O was added and cleanup was done using C18 SPE followed by PGC SPE (described below). As positive control, mucin porcine stomach and bovine fetuin were used. Mucin from porcine stomach was chosen for its complexity in terms of the present mucins. Fetuin was chosen, as it contains well-characterized sialylated O-glycans in high abundance. As negative control mucus medium and water were used.^37,41^

### 2.8 N-Glycan Release Using Enzymatic Treatment

To release the N-glycan, 350 μL of 200 mM ammonium bicarbonate and 65 μL of 2% SDS were added to each sample. The samples were denatured for 10 min at 65 °C and 350 rpm in a Thermomixer. The samples were cooled to room temperature, after which 100 μL of 200 mM ammonium bicarbonate, 100 μL of 4% IGEPAL, 1 μL of 1 mg/mL DP5 internal standard, and 15 μL of PNGase F enzyme (100 U/mL) were added. The samples were vortexed and incubated overnight in a Thermomixer set at 37 °C and 300 rpm. After 4 h of incubation, an additional 5 μL of PNGase F enzyme (100 U/mL) was added. The following day, the samples were split in equal parts, and 1000 μL of ice-cold EtOH (−20 °C) was added to each to approximately 75% EtOH end concentration. The samples were stored at −20 °C for 4–5 h before they were centrifuged at 4 °C at 14,000*g* for 20 min. Afterward, the supernatant was collected and dried in a SpeedVac. The samples were then redissolved in 500 μL H_2_O, and cleanup was done by PGC SPE (described below). As positive controls, mucin porcine stomach and human IgG were used. Mucin from porcine stomach was chosen for its diversity in glycans present. Human IgG was chosen as it contains well-characterized N-glycans in high abundance, both sialylated and nonsialylated. As negative control, mucus medium and water were used.^42,43^

### 2.9 Reversed Phase C18 and Porous Graphitized Carbon Solid Phase Extraction Cleanup

For the O-glycan samples, C18 SPE combined with PGC SPE was performed; for the N-glycan samples, only PGC SPE was used. In short, C18 cartridges were equilibrated using 3 and 1 mL MeOH and 3 and 1 mL H_2_O subsequently. Then, samples were loaded on the column and left for 2 min, and then the flow-through was collected. Washing was performed 3× with 0.5 mL H_2_O and 1x with 0.5 mL H_2_O + 0.1% TFA, and washings were collected. PGC cartridges were equilibrated with 3 and 1 mL of 80% ACN + 0.1% TFA, followed by 3 and 1 mL of H_2_O. For the O-glycan samples, the collected flow-through from the C18 SPE was loaded onto the cartridges, and for the N-glycan samples, the samples were loaded. The samples were left on the column for 2 min. Then, washing was performed 4× with 0.5 mL water, followed by elution using 250 μL 10% ACN, 350 μL 20% ACN, 450 μL 40% ACN + 0.1% TFA, and 200 μL 60% ACN + 0.1% TFA. The eluates were collected and dried using a SpeedVac. For the O-glycan samples, borates were removed by addition of 75 μL MeOH + 0.1% acetic acid, drying, and addition of 2 × 75 μL MeOH and drying. The dried samples were stored at −20 °C until analysis.^44^

### 2.10 MALDI-TOF Mass Spectrometry

The samples were suspended in 100 μL of H_2_O. 1 μL of DP7 (internal standard) was added. The 2,5-DHB MALDI matrix solution (1 μL of 20 mg/mL 2,5-DHB with 0.2 mM NaCl in 50% ACN + 0.1% TFA) was spotted manually on a MALDI MTP target plate (Bruker Daltonics, Bremen, Germany), followed by 1 μL of the sample and dried using a hair dryer. MALDI-TOF MS measurements were performed in positive mode on a Autoflex MaX instrument (Bruker Daltonics, Bremen, Germany). Mass calibration was performed before each analysis using maltodextrin (DP1–20). Glycan spectra were generated from the sum-up of 1500 satisfactory shots in 50 shot steps using a hexagonal shot pattern in the *m*/*z* range 440–3000 using a 500 Hz laser (smartbeam-II solid state laser) with laser power between 40–45%. A disadvantage of MALDI-TOF-MS is that no distinction could be made between isomers, as only *m*/*z* information became available, and therefore, the exact structures could not be confirmed. Data processing was performed by using Bruker Daltonics flexAnalysis 3.4 software. Measured masses were manually interpreted using a list of [M + 23]^+^ or reduced [M + 2 + 23]^+^ masses based on published O- and N-glycan structures reported^4,40,45−47^ and by mass search using GlycoWorkBench version 1.1 (developed by the EUROCarbDB initiative; Supporting Information Tables S1 and S2).

### 2.11 PGC-LC-MS/MS Analysis

The samples were suspended in 50 μL of H_2_O and 1 μL of DP7 (internal standard) was added. 2 μL of the sample was injected. Glycans were separated on a Vanquish ultrahigh pressure liquid chromatography system (Thermo Scientific) equipped with a PGC Hypercarb guard column (10 × 2.1 mm, particle size 3 μM, Thermo Scientific) and a PGC Hypercarb analytical column (150 × 2.1 mm, particle size 3 μM, Thermo Scientific). The mobile phases used were H_2_O + 10 mM NH_4_HCO_3_ (mobile phase A) and 40:60H_2_O/ACN + 10 mM NH_4_HCO_3_ (mobile phase B). The glycans were eluted at a flow rate of 200 μL/min using an optimized gradient from 2% B to 60% B in 40 min. The setup was coupled via a HESI source to a Velos Pro ion trap MS (Thermo Scientific). Data acquisition was organized using two scan events. First, full MS scanning was performed in negative mode in profile data type in the mass range 350–1850 *m*/*z*. Second, a dependent scan in negative mode was performed in centroid data type with CID activation, 4 repeat counts, 5 s repeat duration, 50 exclusion list size, 10 s exclusion time, 1 default charge state, 2.0 *m*/*z* activation width, 34 eV normalized collision energy, and 10 ms activation time.^44^ The MS^2^ fragmentation data were used to verify and/or discover the correct structures/isomers of the various glycans wherever possible. Data processing was performed using Thermo Xcalibur Qual Browser version 4.5.445.18 software. Measured masses were manually interpreted using a list of [M – 1]^−^ or reduced [M + 2 – 1]^−^ masses based on published O- and N-glycan structures reported^4,40,45−47^ and by mass search using GlycoWorkBench version 1.1 (Supporting Information Tables S1–S3).

### 2.12 Data Analysis and Glycan Representation

Glycan representation and visualization was accomplished using GlycoWorkBench version 1.1. The glycan structures were represented according to the Symbol Nomenclature for Glycans.^48^ Represented glycan structures were exported and used in the figures for visualization and clearance on the structures identified. First, identified glycans were compared between the samples. Second, the samples were compared based on ratios between these identified glycans. Third, the relative abundance of the glycans was calculated based on the total peak area of identified glycans and reviewed between the samples. Lastly, the peak height and peak area of added internal standards maltopentaose (DP5; added at the start) and maltoheptaose (DP7; added before analysis) were reviewed between each analysis and consequently compared to the peak height and peak area of the other identified glycan peaks. This was used as a method for reproducibility and for semiquantitative purposes.

## 3 Results and Discussion

### 3.1 Optimization of Glycan Release and Glycan Analysis of *In Vitro* Produced Mucus by HT29-MTX-E12 Cells under SWMS

**Figure 2:**
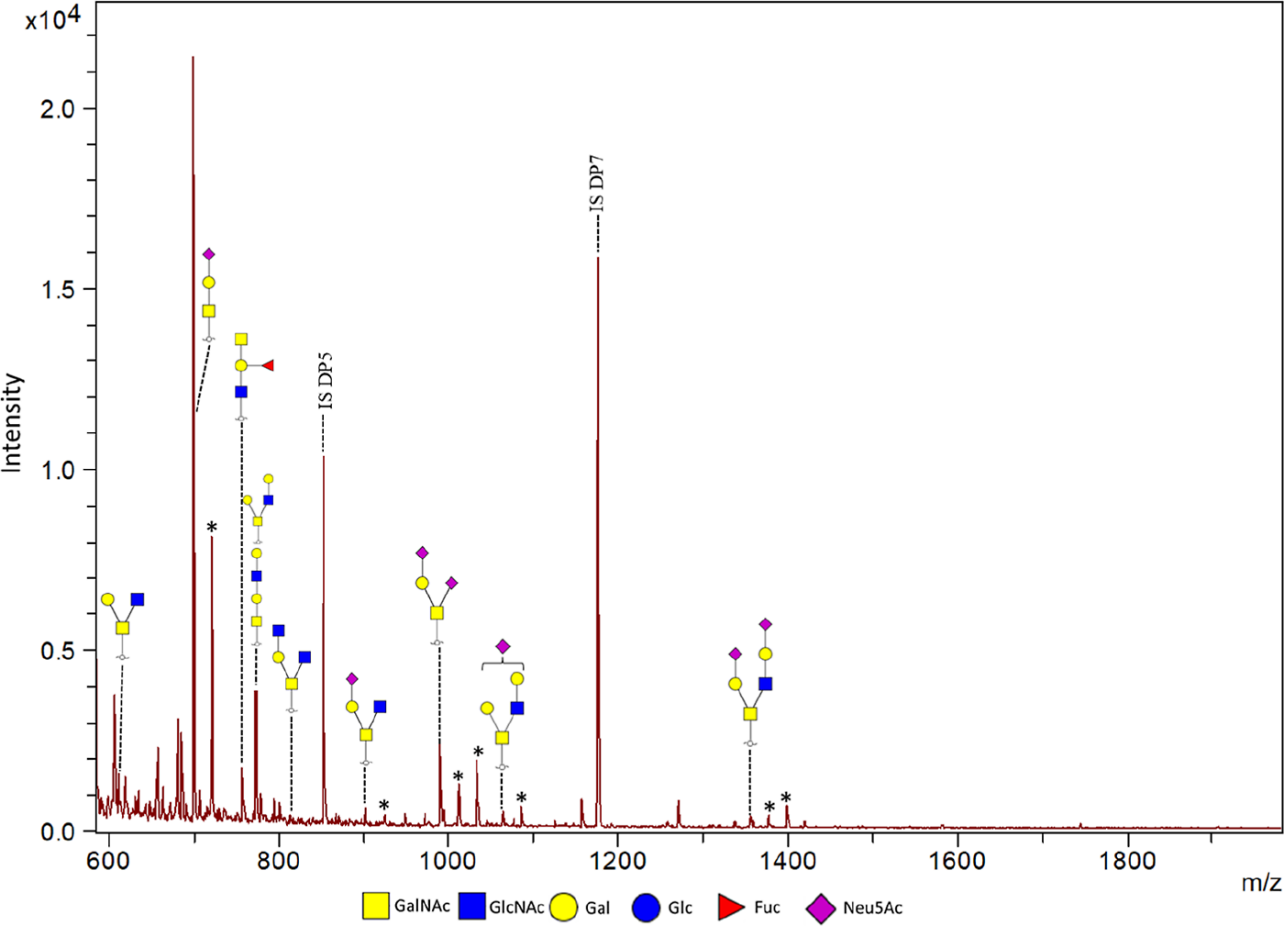
MALDI-TOF mass spectrum of O-glycans released from *in vitro* mucus produced by HT29-MTX-E12 cells under SWMS. Identified peaks as well as the used internal standards DP5 (added at start) and DP7 (added before analysis) are indicated. All peaks are Na^+^ adducts, while sialylated glycans were also detected as [M – H + 2Na]^+^ and [M – 2H + 3Na]^+^ ions (indicated with an *). The structures shown are an interpretation based on measured mass, structures reported in literature, and structure characterization later performed with PGC-LC-MS/MS as described below.

Protocols for O- and N-glycan release and identification were optimized for the analysis of *in vitro* produced mucus. O-glycans were released using chemical β-elimination and the various steps of the protocol were optimized and adapted to efficiently, reproducibly, and completely release all O-glycans in the samples.^37,40,41,44^ This was achieved using commercial standards porcine stomach mucin type III and bovine serum albumin fetuin (Supporting Information, Figures S2 and S3). To include all mucins (soluble and nonsoluble if present), the mucin isolation protocol as described in this paper was used. To verify that only mucin glycans were measured, the protocol was compared with the mucin extraction protocol, as described by Ringot-Destrez et al.^40^ using *in vitro* produced mucus by HT29-MTX-E12 cells under SWMS (Supporting Information Figure S4). Both procedures yielded similar O-glycan profiles and structures, confirming the presence of only mucin glycans. *In vitro* mucus samples were, first of all, analyzed using MALDI-TOF-MS. It is known that cancer cell lines such as HT29-MTX-E12 contain a high level of sialylation and that they only produce low amounts of core 3 and core 4 structures, which are highly abundant in healthy human intestine.^3,7,49^ Therefore, it was hypothesized that the majority of the identified O-glycans would be sialylated and mainly contain core 1 and core 2 structures but that core 3 and core 4 structures would also be identified.^4,23,49^ Mostly core 1 and core 2 O-glycan structures were observed in the MALDI-TOF-MS results (Figure 2). Additional O-glycan structures, presumed to be core 3 and core 4 sialylated forms, were identified as compared to the O-glycans detected by Ringot-Destrez et al.^40^ in their permethylation-based analysis, which could partly be explained by the use of SWMS in the current study. The performed dot-blot assay testing for the presence of MUC2 and MUC5AC (Figure S5) demonstrated the presence of mainly MUC5AC in the produced *in vitro* mucus.

**Figure 3:**
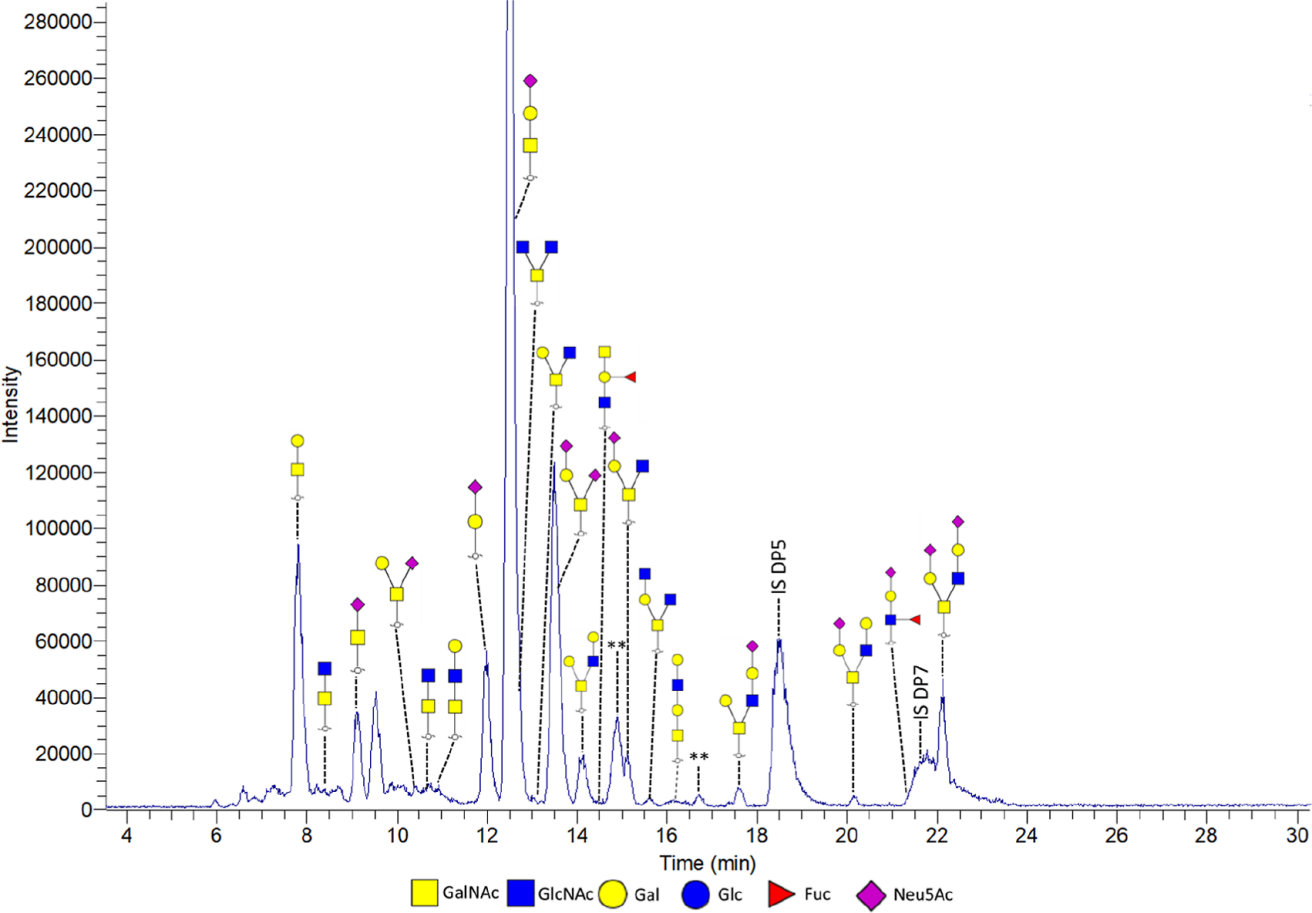
PGC-LC-MS/MS chromatogram of O-glycans released from *in vitro* produced mucus by HT29-MTX-E12 cells grown under SWMS. Identified peaks as well as the used internal standards DP5 (added at start) and DP7 (added before analysis) are indicated. ** Potential artifact, masses could not be confirmed.

The same samples were analyzed using PGC-LC-MS/MS to acquire confirmation and additional structural information regarding the O-glycans (Figure 3). The identified O-glycans using MALDI-TOF-MS were also detected with PGC-LC-MS/MS. PGC-LC-MS/MS analysis not only showed more prominently the sialylated glycans detected by MALDI-TOF MS, but also indicated the presence of additional sialylated structures. Furthermore, PGC-LC-MS/MS provided information regarding smaller structures, which is challenging using MALDI-TOF-MS, as matrix background signals interfere in the lower mass region (mass <550 *m*/*z*). Lastly, information regarding isomeric structures could be acquired based on the fragmentation patterns acquired with PGC-LC-MS/MS (Supporting Information Figure S6). Altogether, a series of mainly sialylated core 1 and core 2 O-glycans but also core 3 and core 4 O-glycans as well as glycan epitope structures were elucidated. Concluding, for O-glycan analysis, MALDI-TOF-MS is convenient for quick screening purposes while PGC-LC-MS/MS is the preferred analysis technique for thorough identification. Therefore, in this paper, only the PGC-LC-MS/MS O-glycan results will be shown. The internal standards DP5/DP7 assisted the semiquantification and enabled monitoring and estimating the amount of glycans per mucin sample. Thus, it can be concluded that, in addition to the data analysis performed by Ringot-Destrez et al.,^40^ we were able to confidently assign all isomeric structures using PGC-LC-MS/MS and to semiquantify the released O-glycans.

**Figure 4:**
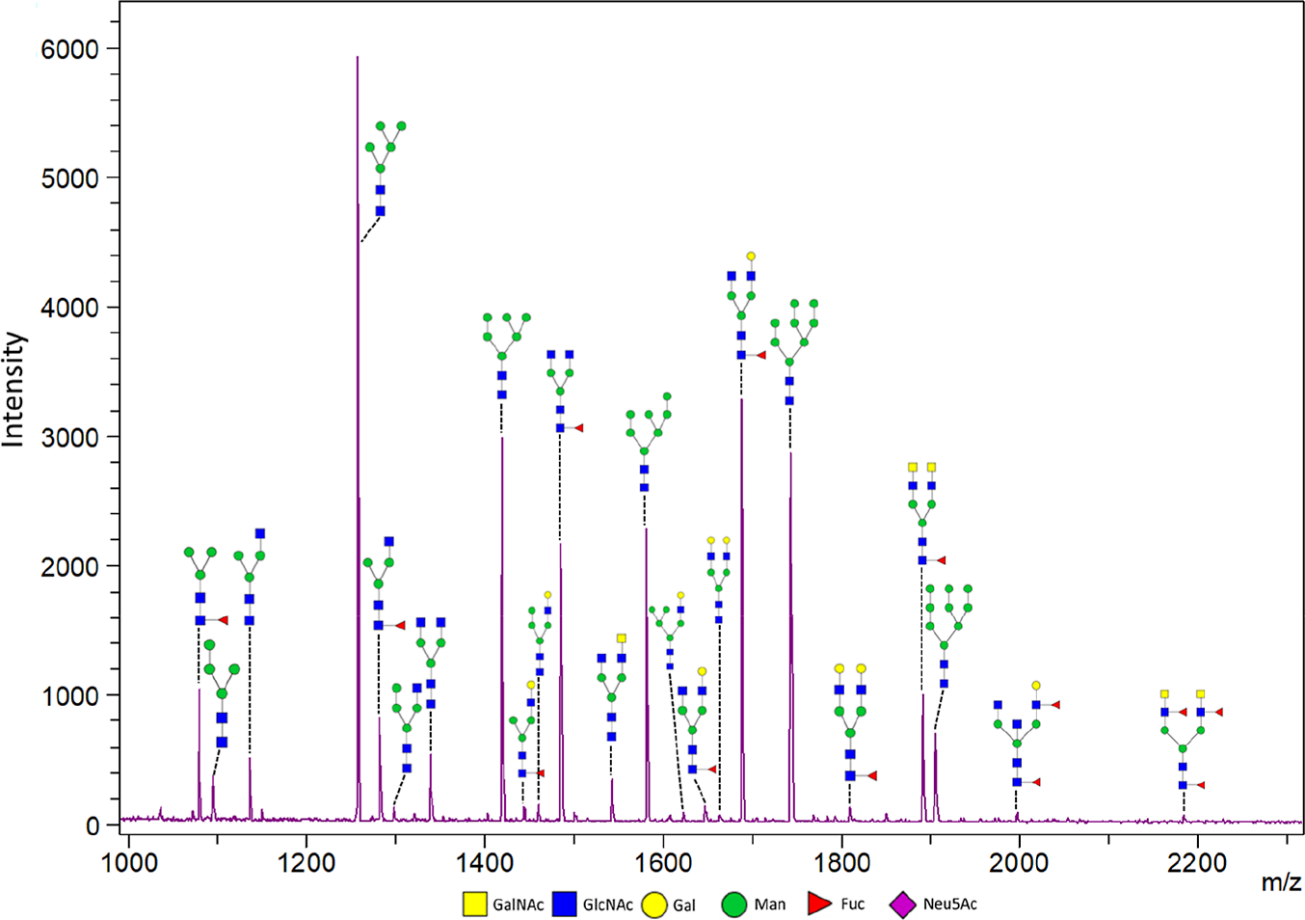
MALDI-TOF mass spectrum of N-glycans released from *in vitro* mucus produced by HT29-MTX-E12 cells under SWMS. Identified peaks and internal standards DP5 (added at the start) and DP7 (added before analysis) are indicated. All of the peaks are Na^+^ adducts. The shown structures are an interpretation based on the measured mass and structures reported in literature. Sialylated N-glycans could not be identified using positive or negative mode MALDI-TOF-MS.

N-glycans are often underrepresented in mucus glycosylation studies and a knowledge gap exists.^10^ This stresses the importance of including N-glycosylation when investigating mucus. The used protocol was based on N-glycan release protocols as described by Holst et al. 2016^42^ and Jansen et al. 2016.^43^ In short, N-glycans were released using enzymatic digestion, and the various steps of the protocol were optimized and adapted to efficiently, reproducibly, and completely release all N-glycans in the samples.^41,42^ This was accomplished using commercial standards human IgG antibody and porcine stomach mucin type III (Supporting Information, Figures S7–S9). *In vitro* mucus samples were first analyzed using MALDI-TOF-MS. Mostly, high mannose N-glycans were abundant (Figure 4) but various complex N-glycans were also identified.^50,51^ Initially, N-glycans are mannose- and glucose-rich, after which the glucose residues are removed in the endoplasmatic reticulum (ER). Then, the structures are trimmed further by specific mannosidases in the Golgi and extended again by modification via specific glycosyltransferases.^52,53^ This explains the diversity observed in high-mannose- and complex-type N-glycans. As expected, the intensity and quantity of the N-glycans was lower than the intensity of the measured O-glycans.^5,54^ With MALDI-TOF-MS, no sialylated N-glycan structures could be elucidated using the employed methods.

**Figure 5:**
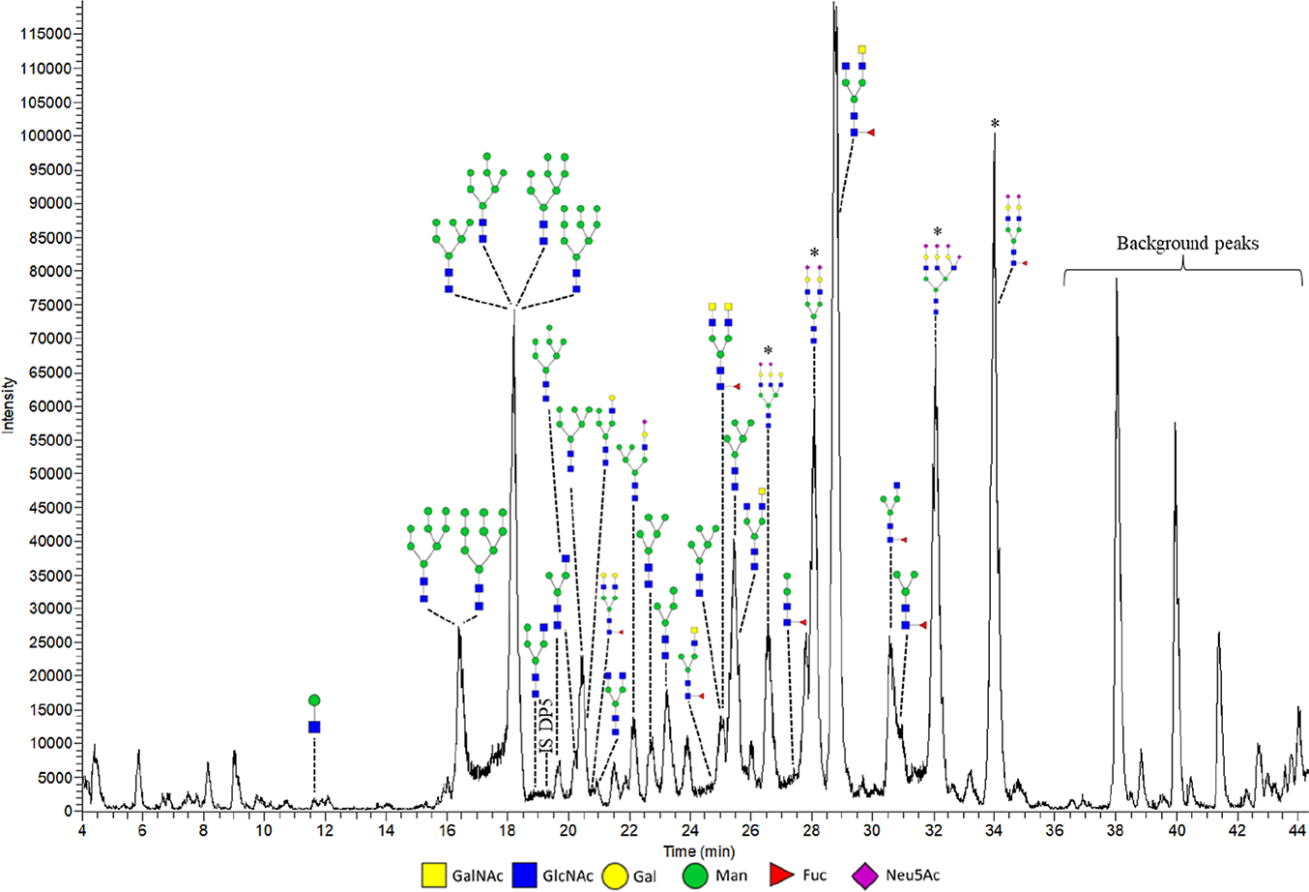
PGC-LC-MS/MS chromatogram of N-glycans released from *in vitro* produced mucus by HT29-MTX-E12 cells under SWMS. Identified peaks as well as the used internal standard DP5 (added at start) are indicated. * peaks originated from the sample matrix or sample preparation.

The same samples were analyzed using PGC-LC-MS/MS to acquire confirmation and additional structural information regarding the N-glycans (Figure 5). The majority of the N-glycan structures identified were similar for both MS techniques; however, using PGC-LC-MS/MS, some of the lowest abundant N-glycans and the higher mass N-glycans could not be identified. It is possible that they bind too strongly to the column, that the mass range of the MS proves insufficient, or that the abundance is below the limit of detection. On the other hand, identification of sialylated N-glycan structures was proven feasible using PGC-LC-MS/MS. The abundance of sialylated N-glycans was low. Unfortunately, the fragmentation patterns obtained for the glycans with PGC-LC-MS/MS were not always conclusive regarding the structure, and in some cases, the fragmentation spectra were too low abundant. Concluding, for N-glycan analysis, PGC-LC-MS/MS is necessary to identify sialylated structures, while MALDI-TOF-MS shows a broader range and additional identified N-glycans. Derivatization could be considered for future work to enable sialylated N-glycan detection with MALDI-TOF-MS.^55,56^ With the current workflow, the use of MALDI-TOF-MS has the preference over PGC-LC-MS/MS, and in this paper, only the MALDI-TOF-MS N-glycan results will be discussed. If PGC-LC-MS/MS analysis revealed the presence of other (sialylated) glycan structures, then this will be indicated.

### 3.2 Variation in Identified O-Glycans and O-Glycan Ratios between Four Separately Produced *In Vitro* Mucus Batches

**Figure 6:**
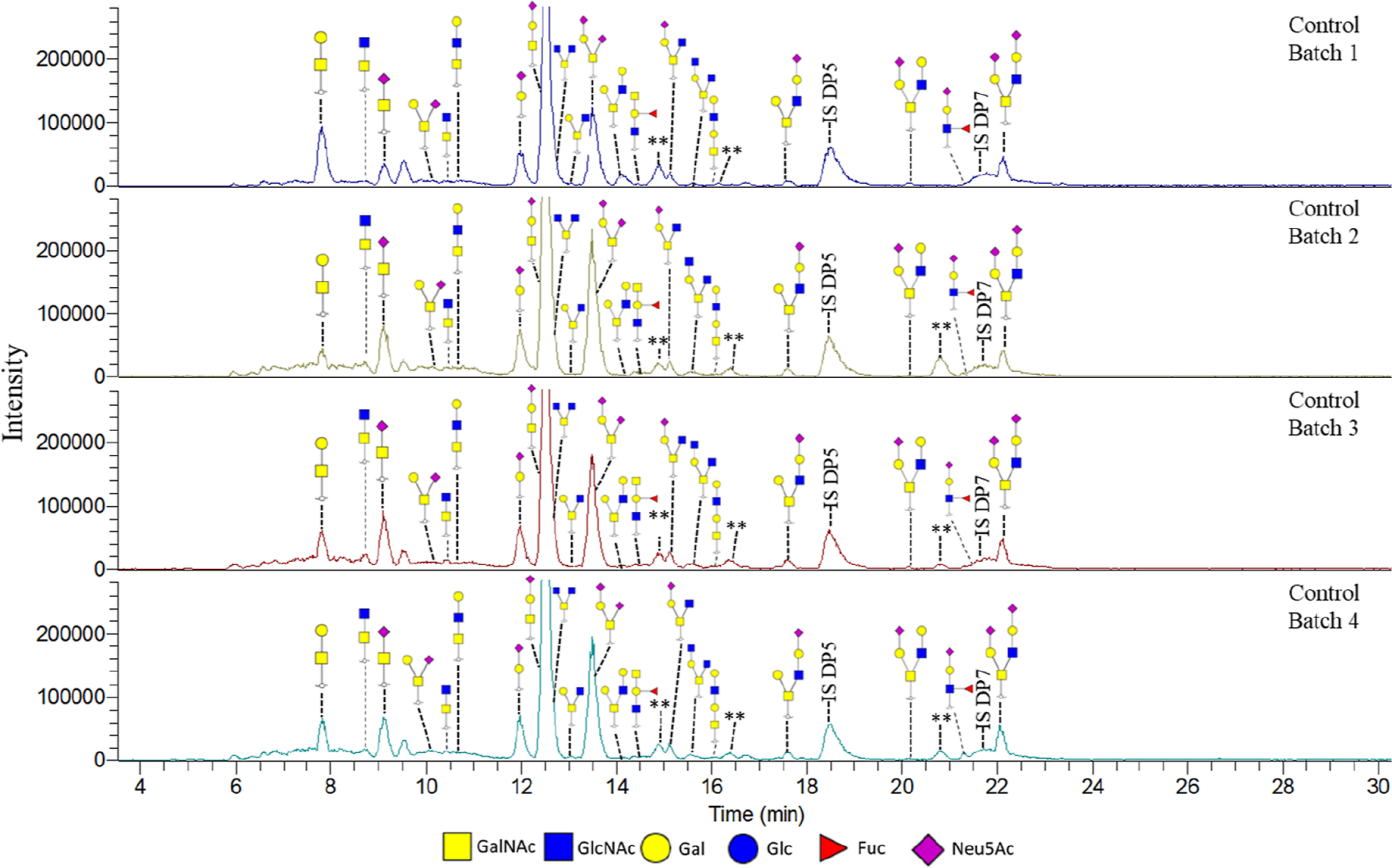
PGC-LC-MS/MS chromatogram of O-glycans released from four individually grown batches of *in vitro* produced mucus by HT29-MTX-E12 cells under SWMS. ** potential artifact, masses could not be confirmed.

The biological variation in *in vitro* produced mucus by HT29-MTX-E12 cells was evaluated using four separately produced *in vitro* mucus batches and measuring the most prominently present O-glycans using MALDI-TOF-MS (data not shown) and PGC-LC-MS/MS.^40^ The structures of the identified O-glycans were similar between the four batches, and also the amount of glycan per mucin sample matched, as demonstrated by the same intensity of the internal standard peak in the various profiles (Figure 6 and Table 1). A certain level of variance was to be expected as this research includes working with cell systems and different batches of samples.^57^ Batch 1 did express larger differences compared with the other three batches in relative abundance of certain identified glycans, especially GalNAc-Gal. The mucin production depends on many different factors, which could partly explain this result. Furthermore, natural variances in the cell growth and glycan production and perhaps a level of degradation could be other factors of influence. Apart from this deviation, the relative areas and glycan ratios between the batches matched well with each other. Therefore, the complete optimized workflow from producing *in vitro* mucus toward glycan analysis is reproducible and contains minimal variation. This setup allowed for the study of mucin glycosylation behavior under different *in vitro* conditions, as will be discussed in the next paragraphs.^20,40^

**Table 1:**
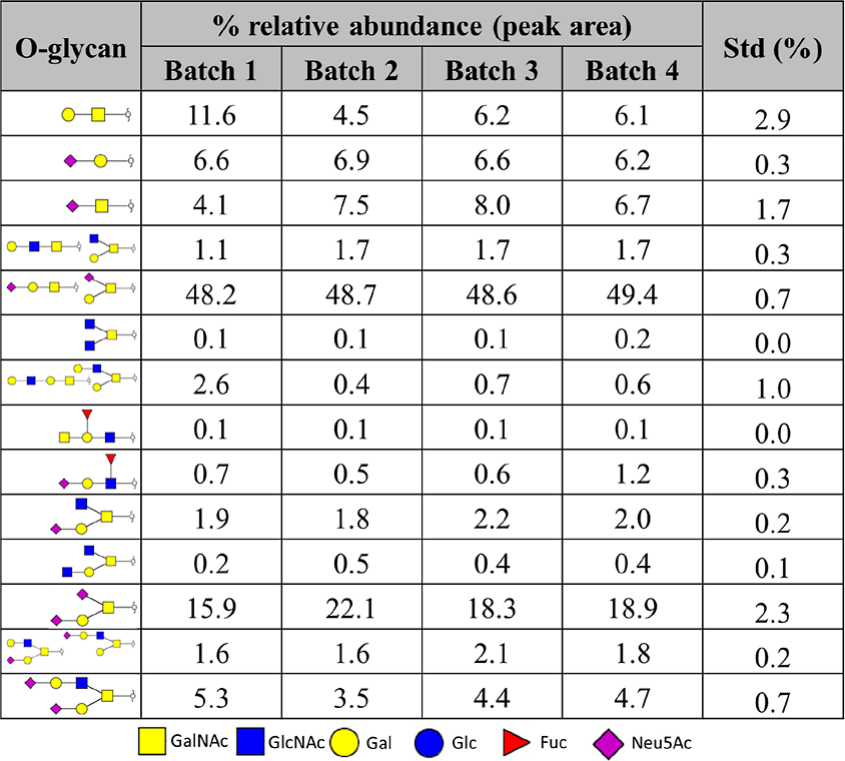
Relative Abundance and Standard Deviation of Identified O-Glycans Released from *In Vitro* Produced Mucus Obtained from Four Individual Batches of HT29-MTX-E12 Cells Grown under SWMS

### 3.3 Analysis of O-Glycans Extracted from *In Vitro* Produced Mucus by HT29-MTX-E12 Cells under SWMS Exposed to Different Concentrations of Pasteurized Bacterium *A. muciniphila*

**Figure 7:**
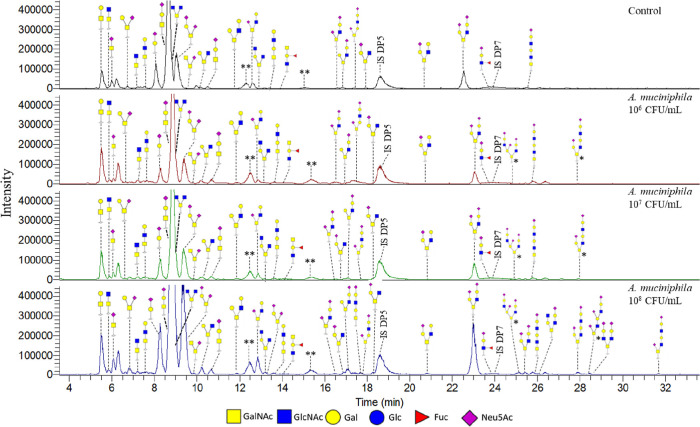
PGC-LC-MS/MS chromatograms of identified O-glycans released from *in vitro* produced mucus by HT29-MTX-E12 cells under SWMS exposed to 10^6^, 10^7^, and 10^8^ cfu/mL pasteurized bacteria *A. muciniphila*. Isomeric structures indicated with * could not be fully verified as the available fragmentation data was not sufficient to fully determine the structure. ** Potential artifact masses could not be confirmed.

To study the effect of *A. muciniphila* on glycosylation, the production of *in vitro* mucus by HT29-MTX-E12 cells was performed in the presence of different concentrations of pasteurized *A. muciniphila*. A prominent effect was seen on the identified O-glycans as well as on the ratios of the O-glycans after analysis with MALDI-TOF-MS (data not shown) and PGC-LC-MS/MS (Figure 7, Table S4). The concentration of 10^6^ cfu/mL of *A. muciniphila* (lowest) showed a glycan profile most similar to the control in terms of identified O-glycans and ratios between O-glycans. At the highest concentration of 10^8^ cfu/mL *A. muciniphila*, additional core 3 and core 4 O-glycan structures were observed as well as variation in the ratios between the O-glycans. Again, results were reproducible, and the changes were caused by the different conditions. So, pasteurized *A. muciniphila* can influence glycosylation as was demonstrated by more expressed O-glycans and more variation in O-glycans initiated by intermediate changes in the concentration of pasteurized *A. muciniphila*. This, again, supports the previous statement that pasteurized bacteria can influence the O-glycosylation patterns of mucus. While these results do not yet give insight into how the O-glycosylation of mucins is regulated by (pasteurized) bacteria, it does stress the importance of the relationship between mucin glycosylation and the intestinal microbiota. Furthermore, it highlights the potential of utilizing *in vitro* produced mucus as a model to study glycosylation and its properties in more detail.

### 3.4 Analysis of O-Glycans and N-Glycans Extracted from *In Vitro* Produced Mucus by HT29-MTX-E12 Cells under SWMS Exposed to Pasteurized Bacteria *A. muciniphila*, *R. gnavus*, and *B. fragilis*

**Figure 8:**
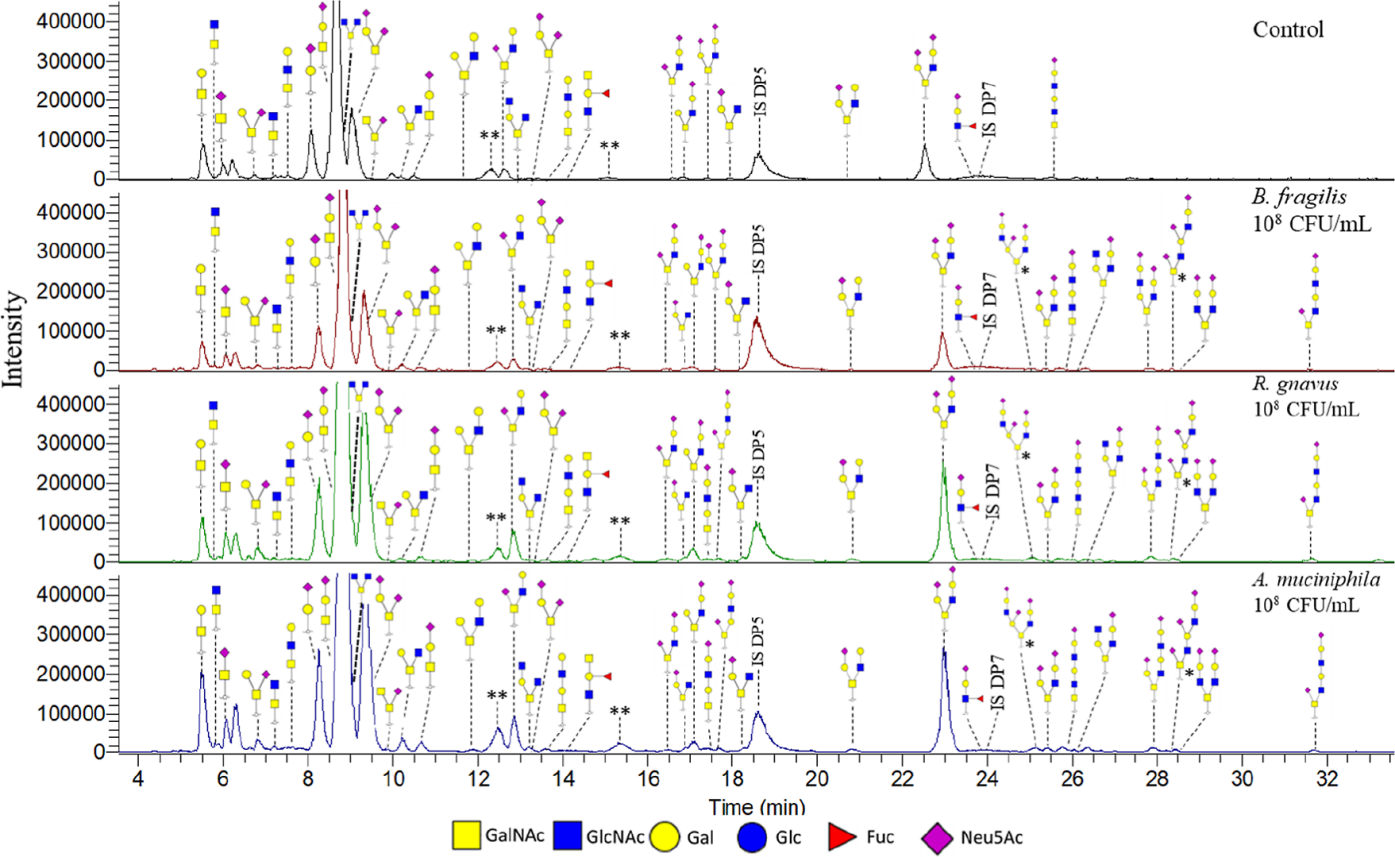
PGC-LC-MS/MS chromatograms of identified O-glycans released from *in vitro* produced mucus by HT29-MTX-E12 cells under SWMS unexposed and exposed to 10^8^ cfu/mL pasteurized bacteria *B. fragilis*, *R. gnavus*, and *A. muciniphila*. * structure could not be fully verified based on available fragmentation data. ** potential artifact, masses could not be confirmed.

To study the effect of specific mucus-associated bacteria on the glycosylation, the production of *in vitro* mucus by HT29-MTX-E12 cells was performed in the presence of pasteurized bacteria *A. muciniphila*, *R. gnavus*, or *B. fragilis*. Again, especially longer and more complex core 3 and core 4 O-glycan structures were identified compared to the unexposed control (Figure 8, Table S5). Additional core 2 O-glycans were also identified. The identified O-glycans were comparable between the three tested pasteurized bacteria expect for one additional O-glycan (RT 17.5 min) identified after exposure to *A. muciniphila* and *R. gnavus*. Furthermore, the ratios between the identified O-glycans after exposure of the cells to the different bacteria expressed subtle differences. These results suggest that the effects are dependent on the pasteurized bacteria. When bacteria are pasteurized, they are inactivated, but the cell walls stay intact. The external environment of the cell, including pili and fimbriae as well as bacterial components such as peptidoglycans, LPS, and exopolysaccharides, can still interact.^58^ These components could (indirectly) stimulate the (enzymatic) processes involved in glycosylation in the ER and Golgi of the growing cells, influencing the produced mucus. This was also discussed in studies regarding the beneficial effects of pasteurized compared to nonpasteurized exposure to *A. muciniphila*.^33,34^ It was hypothesized that the bacteria could have a different effect on the glycosylation patterns, as each of these intestinal microbes has its own outer membrane composition. As LPS is the major component of the outer membrane of Gram-negative bacteria and therefore will be present in all three selected bacteria, the effects were difficult to predict. Since an increase was observed in sialylated core 2, core 3, and core 4 structures, it could be speculated that the pasteurized bacteria influence the glycosyltransferases (GTs) necessary for the biosynthesis of those structures or the genes involved in the GT expression. GTs involved in biosynthesis of core 2, core 3, and core 4 structures include GT31, GT14, GT7, and GT29.^59^ An explanation for the minor differences could be that bacterial characteristics had a minimal differential effect, the concentrations of pasteurized bacteria were not high enough to show major differences, the cell line used was less sensitive to the tested variables, the *in vitro* model is not representative enough, or the pasteurization subdued individual differences. All three selected bacteria are known as mucin degraders, *R. gnavus* and *A. muciniphila* are specialists, while *B. fragilis* is a generalist.^60^ They all are capable of expressing glycosyl hydrolases such as fucosidases, sialidases, β-galactosidases, β-GlcNAcases, and α-GalNAcases, which are necessary for mucin degradation and utilization to thrive in the mucus niche.^60^ As pasteurization inactivates these enzymes, their activity was not expected to influence the glycosylation outcome.

**Figure 9:**
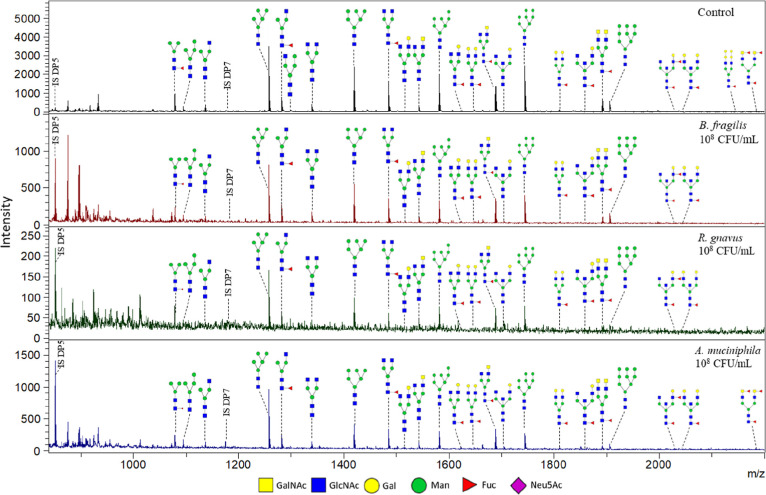
MALDI-TOF mass spectra of identified N-glycans released from *in vitro* produced mucus by HT29-MTX-E12 cells under SWMS unexposed and exposed to 10^8^ cfu/mL pasteurized bacteria *B. fragilis*, *R. gnavus*, and *A. muciniphila*. Identified peaks and internal standards DP5 (added at start) and DP7 (added before analysis) are indicated. All peaks are Na^+^ adducts. The shown structures are an interpretation based on the measured mass and structures reported in literature. Sialylated N-glycans could not be identified using positive or negative mode MALDI-TOF-MS.

The majority of the N-glycan structures were characterized in both the control sample and the samples exposed to pasteurized bacteria with MALDI-TOF-MS and PGC-LC-MS/MS (data not shown). However, some of the least abundant complex-type N-glycans were barely or not detected in the samples exposed to pasteurized bacteria. This matched well with the observed decrease in overall intensity of the identified N-glycans in the samples exposed to pasteurized bacteria (compared to IS DP5 and DP7). Furthermore, glycan ratios showed variance as well, mainly in the high mannose N-glycans structures (Figure 9, Supporting Information Table S6). The relative abundance of high mannose N-glycans Man8 and Man9 in the samples exposed to pasteurized bacteria was increased. The other high mannose N-glycans seemed to show a slight decrease in the samples exposed to pasteurized bacteria. These results suggest that the N-glycosylation, although less than the O-glycosylation, is also influenced by pasteurized bacteria resulting in less overall abundance of N-glycans and up-regulation or down-regulation of specific high mannose N-glycans.

It should be taken into account that *in vitro* mucus produced by HT29-MTX-E12 cells grown under adapted SWMS in cell culture flasks is not identical to *in vivo* human intestinal mucus.^20^ First of all, mostly MUC5AC was detected instead of MUC2 (Supporting Information Figure S5). Moreover, differences are to be expected in terms of type of core structures, presence of specific isomers, type of branching, and abundance of sialylation or fucosylation.^4,61^ Still, this research clearly shows that *in vitro* mucus, containing similar glycans to those of *in vivo* human intestinal mucus, can be produced and manipulated. Furthermore, this study highlights the potential of pasteurized bacteria. We speculate that bacterial components such as peptidoglycans, LPS, and exopolysaccharides as well as the external environment of the cell can (indirect) interact with the enzymatic processes in glycan production, but future research is needed to investigate which components are responsible for the observed effects on glycosylation. Nevertheless, this study shows that the external environment of the cell can influence the produced mucus as well as the glycosylation patterns and perhaps even stimulate mucin production. All in all, this study convincingly shows that the applied less intrusive and easily accessible approach is highly promising in order to gain a better understanding of the mechanisms between the intestinal microbiota and O-glycosylation as well as N-glycosylation of mucins.

## Supporting Information Available

Spectra and chromatograms of the *in vitro* mucus sample analyzed according to Ringot-Destrez et al. 2018 and reference standards used (porcine stomach mucin and IgG), fragmentation spectra of different analyzed O-glycans, dot-blot scans for MUC2 and MUC5AC, tables containing peak area and relative abundance of O-glycans from *in vitro* mucus samples, and tables containing information regarding the (LC–)MS methods used and information about the samples and sample treatment (PDF)

## Abbreviations

*A. muciniphila*: *Akkermansia muciniphila*
*B. fragilis*: *Bacteroides fragilis*
BHIS: brain heart infusion
CID: collision-induced dissociation
cfu: colony forming unit
ER: endoplasmic reticulum
ECACC: European Collection of Authenticated Cell Cultures
Fuc: fucose
Gal: galactose
GalNAc: *N*-acetylgalactosamine
Glc: glucose
GlcNAc: *N*-acetylglucosamine
HESI: heated electrospray ionization
IGEPAL: octylphenoxypolyethoxyethanol
LPS: lipopolysaccharides
MALDI-TOF-MS: matrix-assisted laser desorption/ionization-time-of-flight mass spectrometry
Maltoheptaose: DP7
Maltopentaose: DP5
MS: mass spectrometry
Neu5Ac: *N*-acetylneuraminic acid
OD: optical density
PGC: porous graphitized carbon
*R. gnavus*: *Ruminoccocus gnavus*
SDS: sodium dodecyl sulfate
SPE: solid phase extraction
SWMS: semi-wet interface with mechanical stimulation
UPLC: ultrahigh performance liquid chromatography
