# Supplementary material for: Revealing Glycosylation Patterns in In Vitro-Produced Mucus Exposed to Pasteurized Mucus-Associated Intestinal Microbes by MALDI-TOF-MS and PGC-LC-MS/MS
Source: J Agric Food Chem. 2024 Jun 27;72(27):15345–56. doi: 10.1021/acs.jafc.4c01401 (PMC11247495; doi:10.1021/acs.jafc.4c01401)
Supplement: Supplementary file 1 — jf4c01401_si_001.pdf [file jf4c01401_si_001.pdf]

Supporting information

Revealing glycosylation patterns in *in vitro* produced mucus exposed to pasteurised mucus-associated intestinal microbes by MALDI-TOF-MS and PGC-LC-MS/MS

Carol de Ram[a], Benthe van der Lugt[c°], Janneke Elzinga[b●], Sharon Geerlings[b], Wilma T. Steegenga[c†], Clara Belzer[b‡], Henk A. Schols[a*‡]

[a] *Laboratory of Food Chemistry, Wageningen University & Research, Bornse Weilanden 9, 6708 WG Wageningen, the Netherlands*

[b] *Laboratory of Microbiology, Wageningen University & Research, Stippeneng 4, 6708 WE Wageningen, the Netherlands*

[c] *Human Nutrition and Health, Wageningen University & Research, Stippeneng 4, 6708 WE Wageningen, the Netherlands*

* Corresponding author.

E-mail address: henk.schols@wur.nl (H.A. Schols)

‡These authors contributed equally to this work.

† Deceased.

°Present address: *Wageningen Food Safety Research, Akkermaalsbos 2, 6708 WB Wageningen, the Netherlands.*

●Present address: *Copenhagen Center for Glycomics, Department of Cellular & Molecular Medicine, University of Copenhagen, DK-2200, Copenhagen, Denmark*

Table of contents

Figure S1. Visualisation of the eight O-glycan core structures that have been identified in mammalian organisms. Core structures 1 and 2 are considered most common. 4
Figure S2. MALDI-TOF mass spectrum of O-glycans released from four separately prepared porcine stomach mucin type III used as method control. Assigned structures could be isomers as well as they have not been properly identified regarding their exact structure. Yellow square = GalNAc, blue square = GlcNAc, yellow circle = Gal, red triangle = Fuc, purple diamond = Neu5Ac. 5
Figure S3. PGC-LC-MS/MS chromatogram of O-glycans released from porcine stomach mucin type III used as method control. Assigned structures could be isomers as well as they have not been properly identified regarding their exact structure. Yellow square = GalNAc, blue square = GlcNAc, yellow circle = Gal, red triangle = Fuc, purple diamond = Neu5Ac. 6
Figure S4. MALDI-TOF mass spectrum of O-glycans released from *in vitro* mucus produced by HT29-MTX-E12 cells using Semi-Wet conditions with Mechanical Stimulation. The mucins were extracted according to the protocol as described by Ringot-Destrez et al. 2018. All peaks are are Na^+ adducts, while sialylated glycans were also detected as $[M\text{-}H\text{+}2Na]^+$ and $[M\text{-}2H\text{+}3Na]^+$ ions (indicated with *). 7
Figure S5. Dot-blot scan for presence of MUC2 (left) and MUC5AC (right) using anti-MUC2 and anti-MUC5AC as primary antibody and anti-mouse as secondary antibody. The top spots are undiluted sample and proceding down each spot is a two times dilution from the previous spot. The samples are *in vitro* produced mucus as used during the manuscript. 8
Figure S6. Example fragmentation spectra of identified O-glycans. The spectra show the different mass peaks which were used for interpretation of the glycan structures. Spectra A-J are from in vitro mucus control batch (figure 7) and spectra K-S are from in vitro mucus A. muciniphila 108 CFU/mL (figure 7). Yellow square = GalNAc, blue square = GlcNAc, blue circle = Glc, yellow circle = Gal, red triangle = Fuc, purple diamond = Neu5Ac. 18
Figure S7. MALDI-TOF mass spectrum of N-glycans released from two separately prepared human IgG used as method control. Yellow square = GalNAc, blue square = GlcNAc, green circle = mannose, yellow circle = Gal, red triangle = Fuc, purple diamond = Neu5Ac. 19
Figure S8. MALDI-TOF mass spectrum of N-glycans released from porcine stomach mucin type III used as method control. Yellow square = GalNAc, blue square = GlcNAc, green circle = mannose, yellow circle = Gal, red triangle = Fuc, purple diamond = Neu5Ac. 20
Figure S9. PGC-LC-MS/MS chromatogram of N-glycans released from human IgG used as method control. Unassigned peaks originate from the sample preparation, sample matrix, or are potential artifacts. Yellow square = GalNAc, blue square = GlcNAc, green circle = mannose, yellow circle = Gal, red triangle = Fuc, purple diamond = Neu5Ac. 21

Table S1. Mass spectrometric information based on MIRAGE guidelines (Kolarich et al. 2013). 22
Table S2. Sample information overview based on MIRAGE guidelines (Struwe et al 2016). 23
Table S3. Liquid chromatographic information of used set-up for the analysis of in vitro produced mucus and standards IgG and porcine stomach mucin type III based on MIRAGE guidelines (Campbell et al. 2019). 24
Table S4. Peak area and calculated relative abundance percentage based on peak area of identified O-glycans released from *in vitro* produced mucus obtained from HT29-MTX-E12 cells grown under Semi-Wet conditions with Mechanical Stimulation exposed to different concentrations of pasteurised *A. muciniphila*. Yellow square = GalNAc, blue square = GlcNAc, yellow circle = Gal, blue circle = Glc, red triangle = Fuc, Purple diamond = Neu5Ac 25
Table S5. Peak area and calculated relative abundance percentage based on peak area of identified O-glycans released from *in vitro* produced mucus obtained from HT29-MTX-E12 cells grown under Semi-Wet conditions with Mechanical Stimulation exposed to different pasteurised bacteria *B. fragilis*, *R. gnavus*, and *A. muciniphila*

at concentration of 10E6 CFU/mL. Yellow square = GalNAc, blue square = GlcNAc, yellow circle = Gal, blue circle = Glc, red triangle = Fuc, Purple diamond = Neu5Ac 26
Table S6. Peak area and calculated relative abundance percentage based on peak area of identified N-glycans released from *in vitro* produced mucus obtained from HT29-MTX-E12 cells grown under Semi-Wet conditions with Mechanical Stimulation exposed to different pasteurised bacteria *B. fragilis*, *R. gnavus*, and *A. muciniphila* at concentration of 10E6 CFU/mL. Yellow square = GalNAc, blue square = GlcNAc, yellow circle = Gal, blue circle = Glc, red triangle = Fuc, Purple diamond = Neu5Ac 27

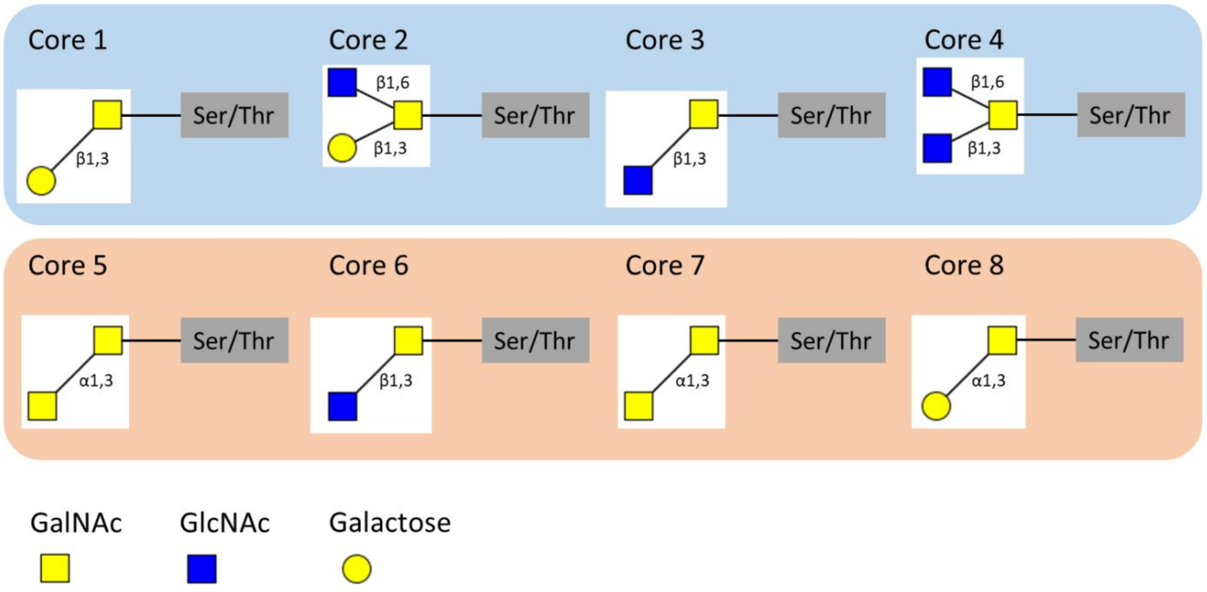

Figure S1. Visualisation of the eight O-glycan core structures that have been identified in mammalian organisms. Core structures 1 and 2 are considered most common.

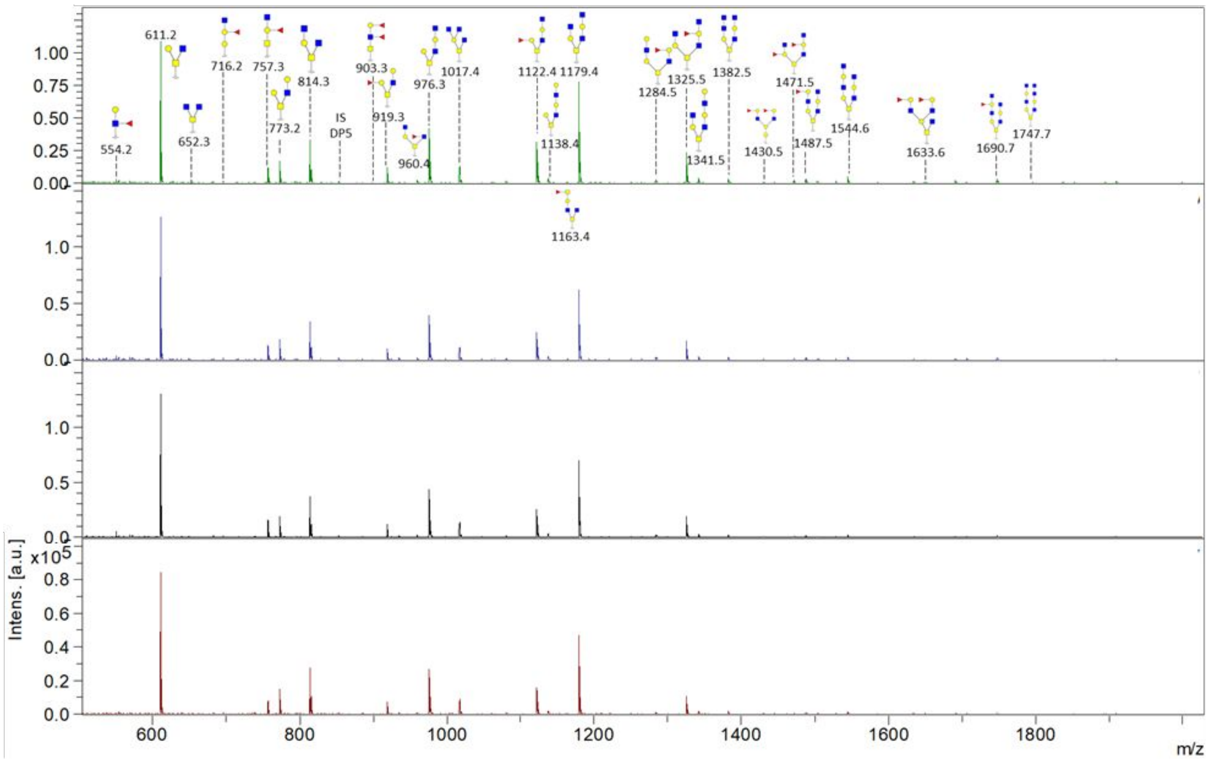

Figure S2. MALDI-TOF mass spectrum of O-glycans released from four separately prepared porcine stomach mucin type III used as method control. Assigned structures could be isomers as well as they have not been properly identified regarding their exact structure. Yellow square = GalNAc, blue square = GlcNAc, yellow circle = Gal, red triangle = Fuc, purple diamond = Neu5Ac.

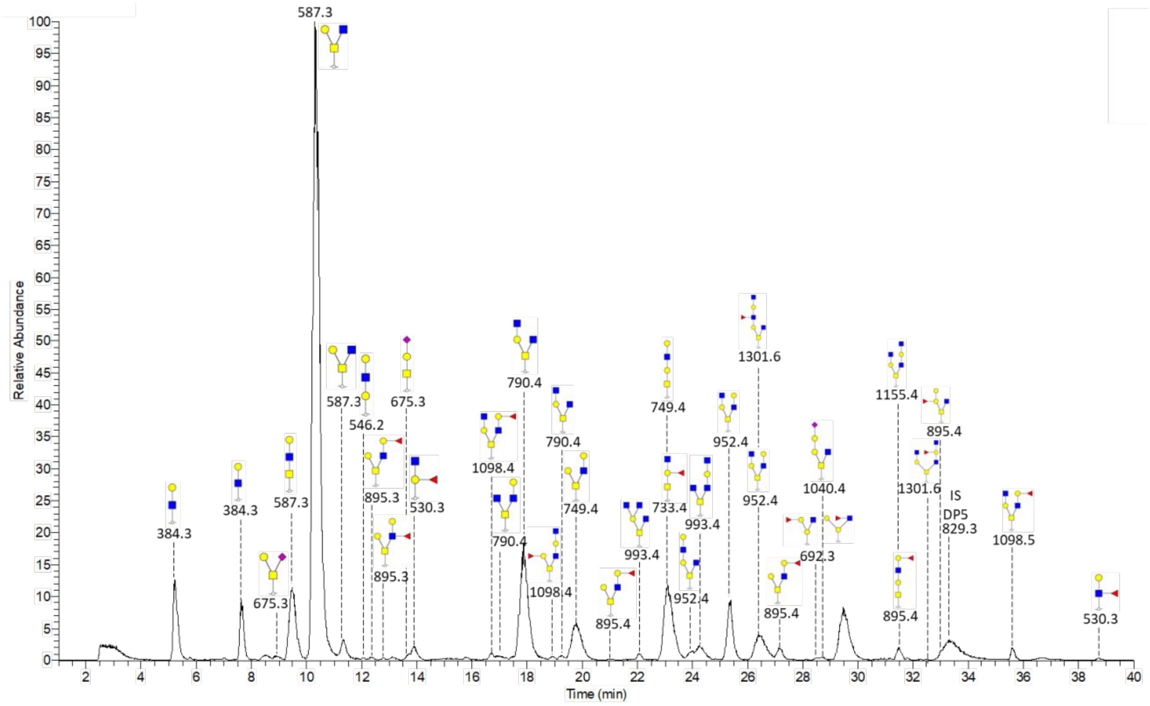

Figure S3. PGC-LC-MS/MS chromatogram of O-glycans released from porcine stomach mucin type III used as method control. Assigned structures could be isomers as well as they have not been properly identified regarding their exact structure. Yellow square = GalNAc, blue square = GlcNAc, yellow circle = Gal, red triangle = Fuc, purple diamond = Neu5Ac.

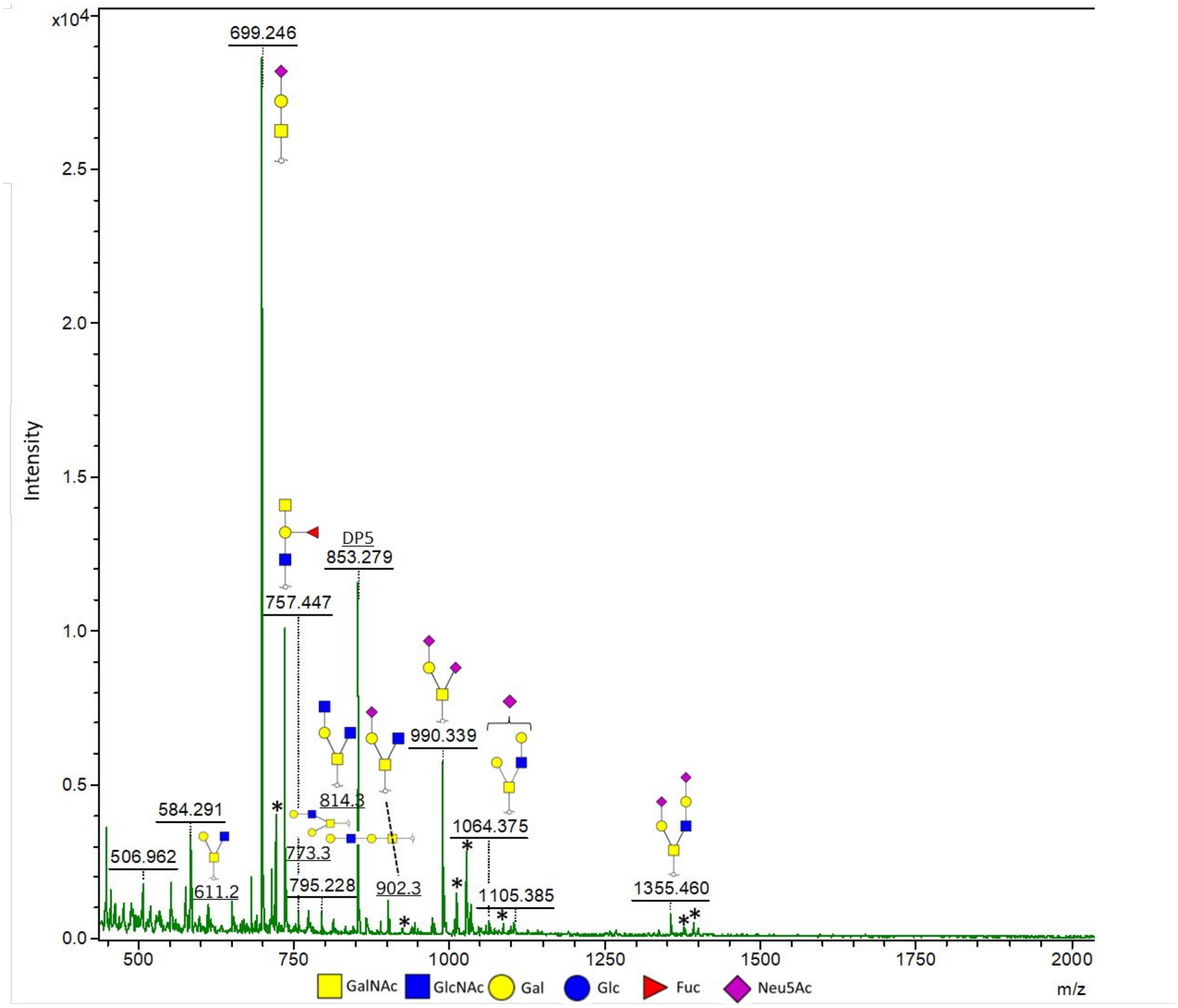

Figure S4. MALDI-TOF mass spectrum of O-glycans released from *in vitro* mucus produced by HT29-MTX-E12 cells using Semi-Wet conditions with Mechanical Stimulation. The mucins were extracted according to the protocol as described by Ringot-Destrez et al. 2018. All peaks are are Na^+ adducts, while sialylated glycans were also detected as $[M-H+2Na]^+$ and $[M-2H+3Na]^+$ ions (indicated with *).

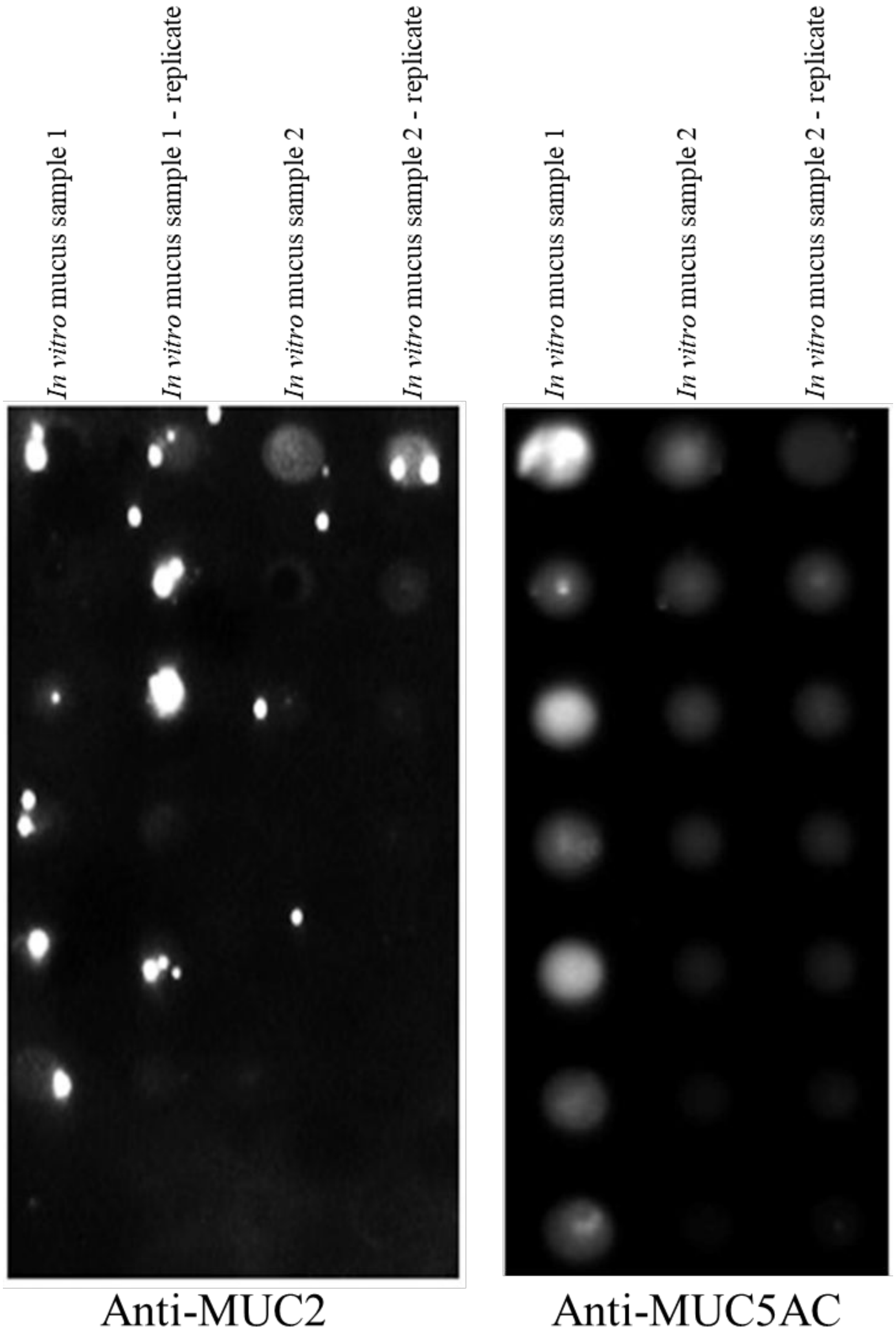

Figure S5. Dot-blot scan for presence of MUC2 (left) and MUC5AC (right) using anti-MUC2 and anti-MUC5AC as primary antibody and anti-mouse as secondary antibody. The top spots are undiluted sample and proceding down each spot is a two times dilution from the previous spot. The samples are *in vitro* produced mucus as used during the manuscript.

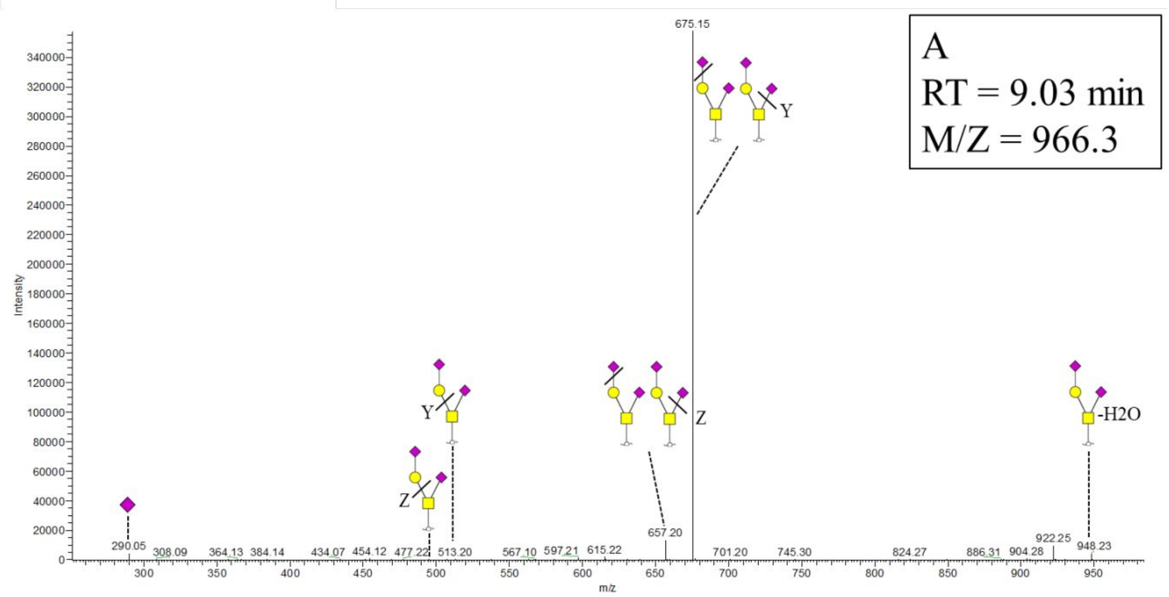
A
RT = 9.03 min
M/Z = 966.3
675.15
Y
Z
-H2O
290.05
513.20
657.20
948.23
922.25
Intensity
m/z

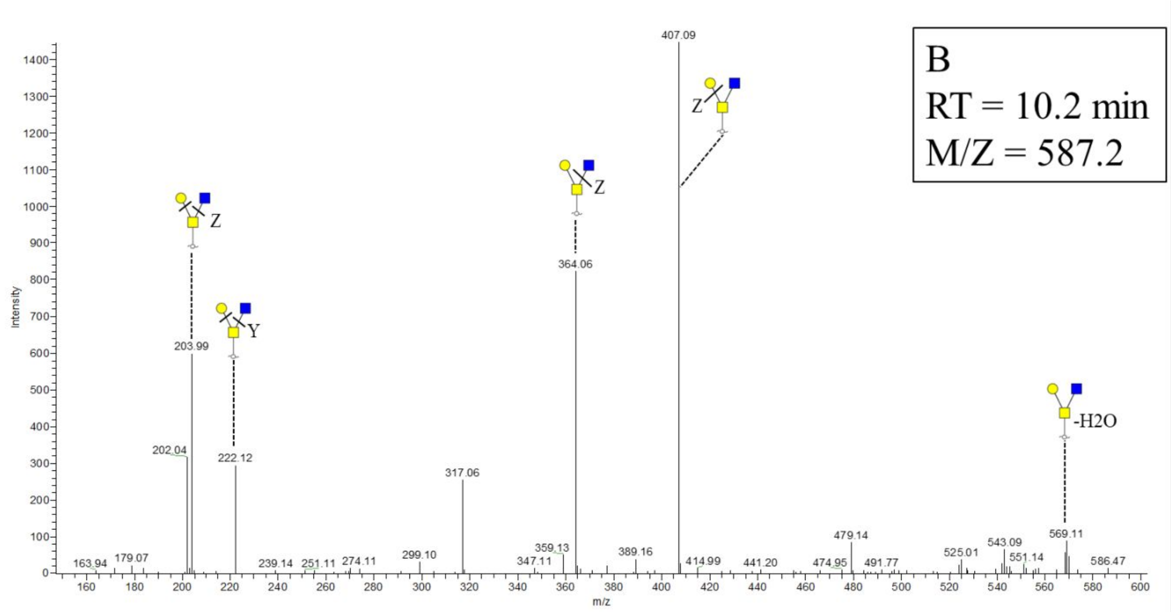
B
RT = 10.2 min
M/Z = 587.2
407.09
364.06
203.99
222.12
202.04
317.06
569.11
Z
Y
-H2O
Intensity
m/z

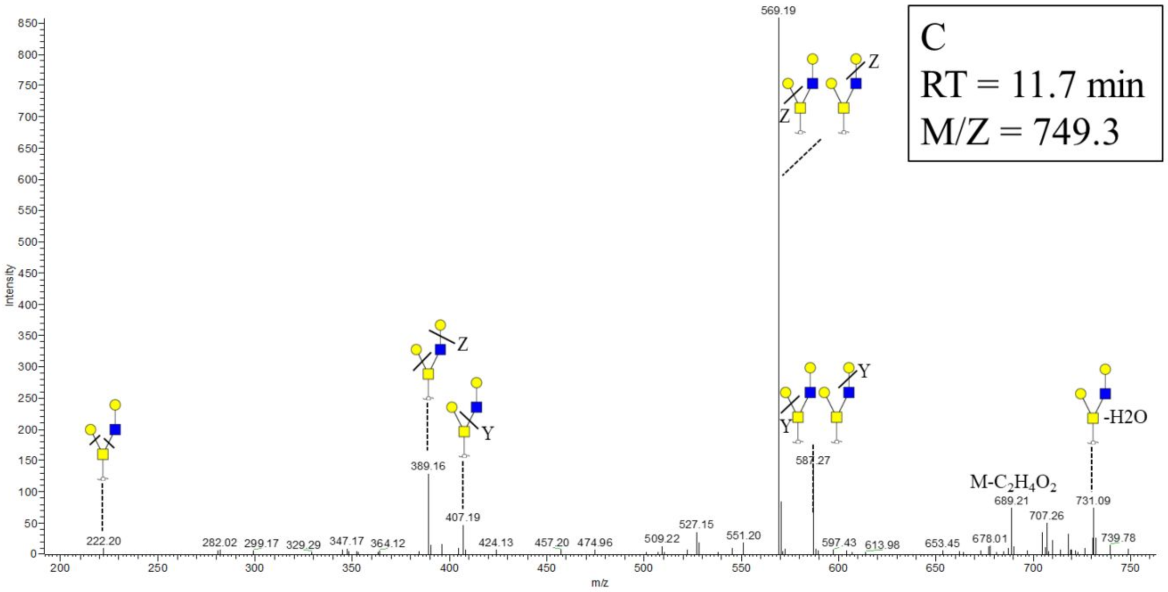
C
RT = 11.7 min
M/Z = 749.3
Intensity
m/z
569.19
389.16
407.19
222.20
587.27
M-C2H4O2
689.21
707.26
731.09
739.78
-H2O
Z
Y

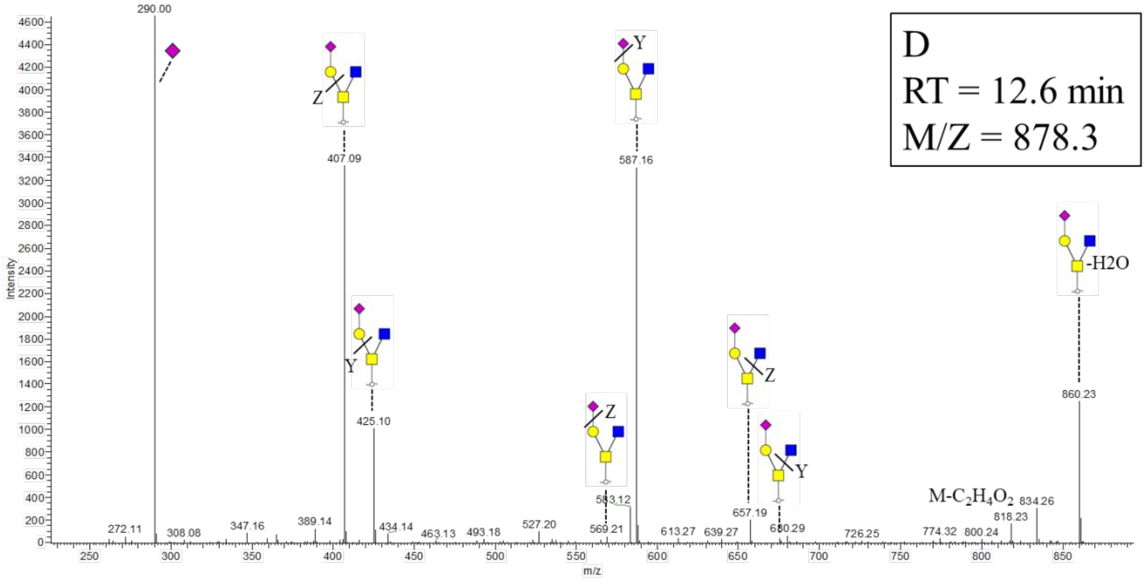
D
RT = 12.6 min
M/Z = 878.3
Intensity
m/z
290.00
407.09
425.10
587.16
583.12
657.19
680.29
860.23
M-C2H4O2
818.23
834.26
-H2O
Z
Y

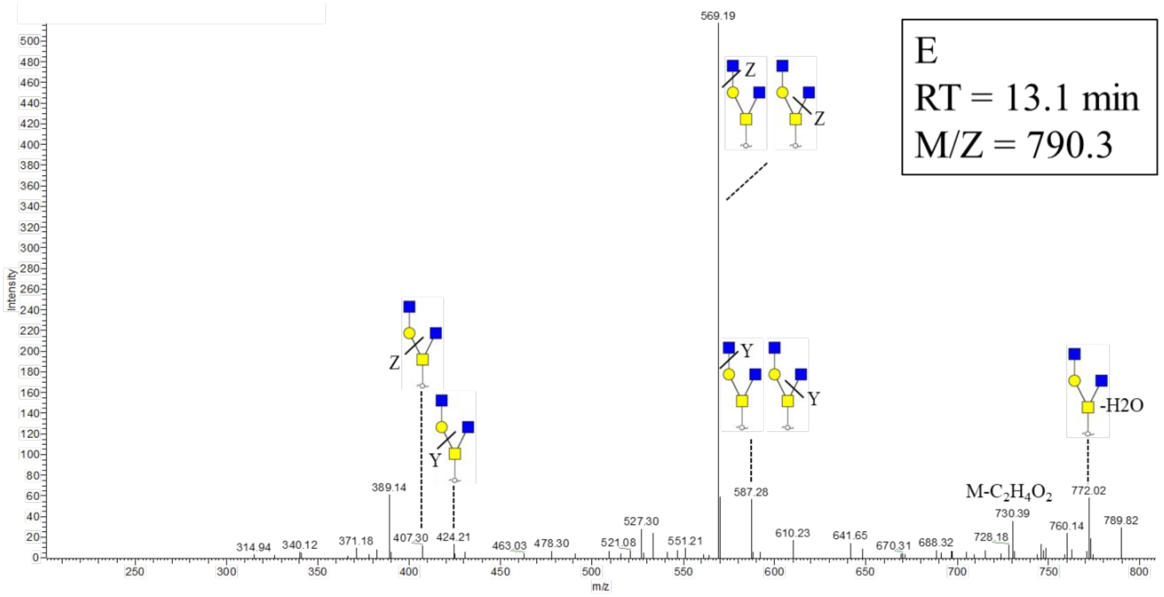
E
RT = 13.1 min
M/Z = 790.3
569.19
587.28
389.14
407.30
424.21
527.30
772.02
789.82
730.39
760.14
M-$C_2H_4O_2$
-H2O
Intensity
m/z

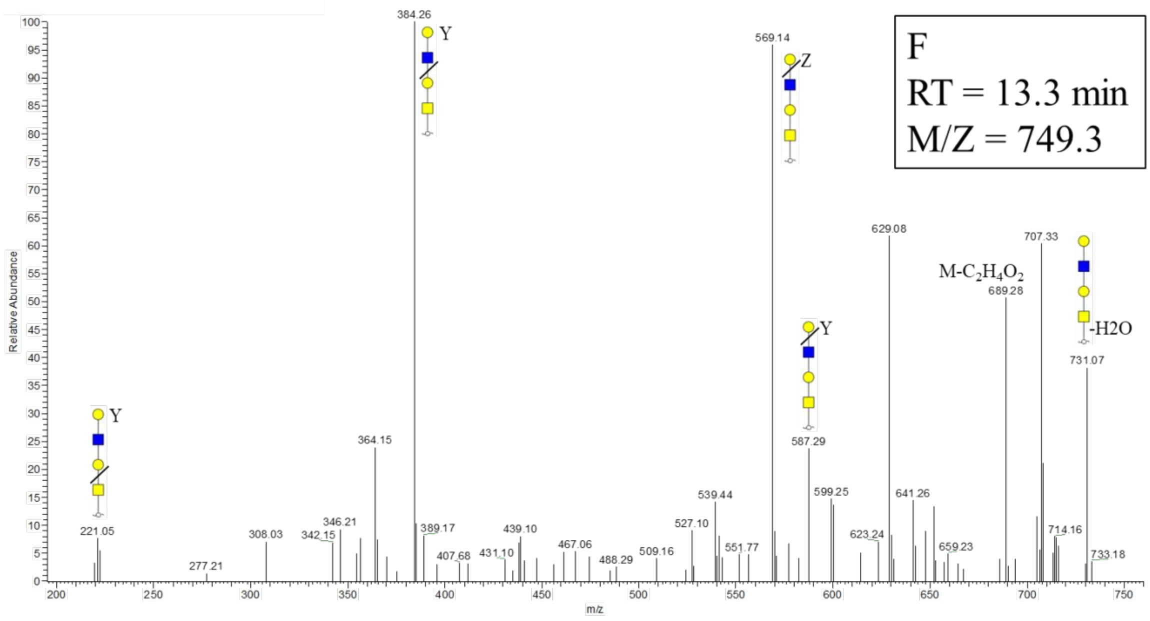
F
RT = 13.3 min
M/Z = 749.3
384.26
569.14
629.08
707.33
689.28
731.07
587.29
364.15
221.05
M-$C_2H_4O_2$
-H2O
Relative Abundance
m/z

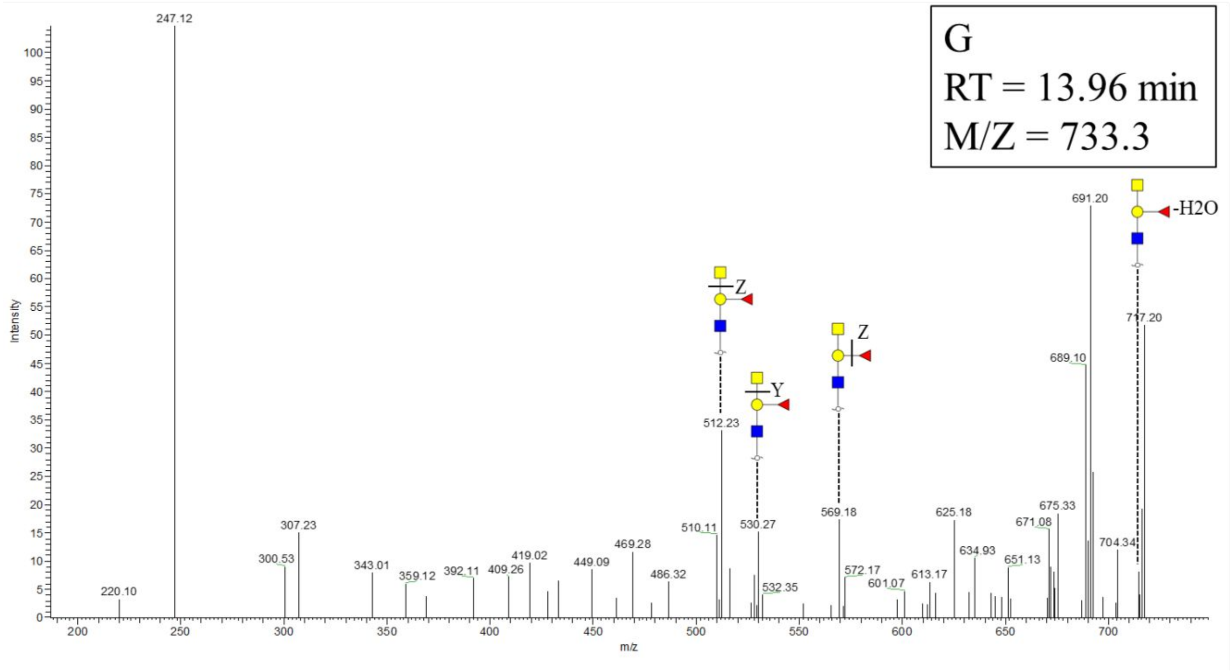
G
RT = 13.96 min
M/Z = 733.3
-H2O
Z
Y
Intensity
m/z

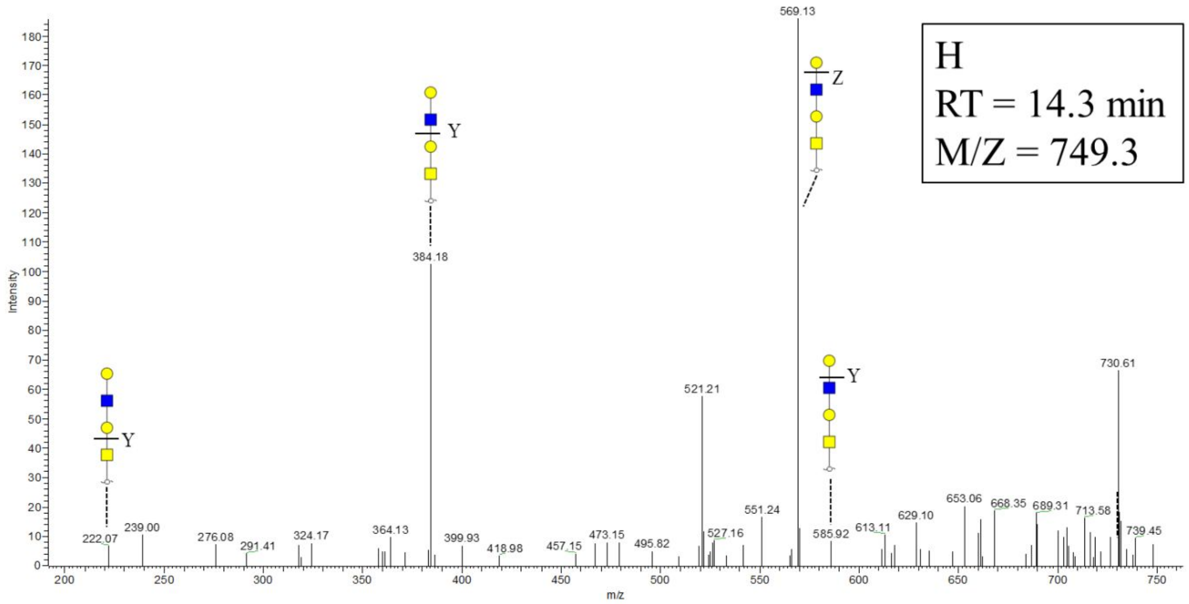
H
RT = 14.3 min
M/Z = 749.3
Z
Y
Intensity
m/z

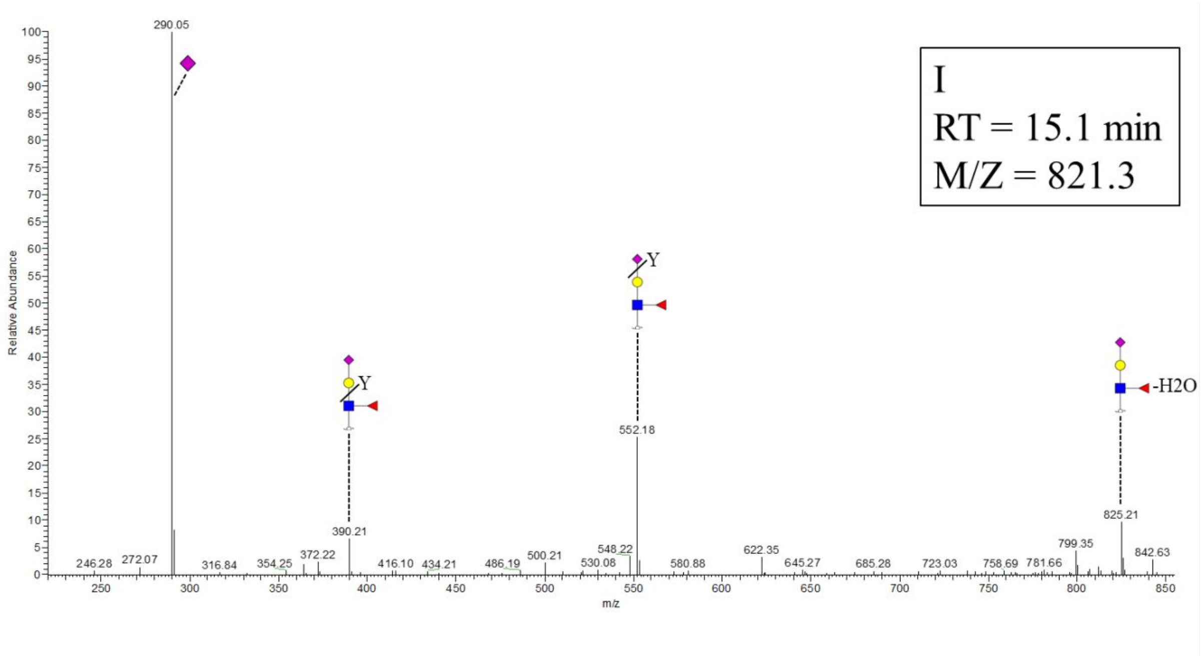
I
RT = 15.1 min
M/Z = 821.3
Relative Abundance
m/z
290.05
390.21
552.18
825.21
-H2O
Y

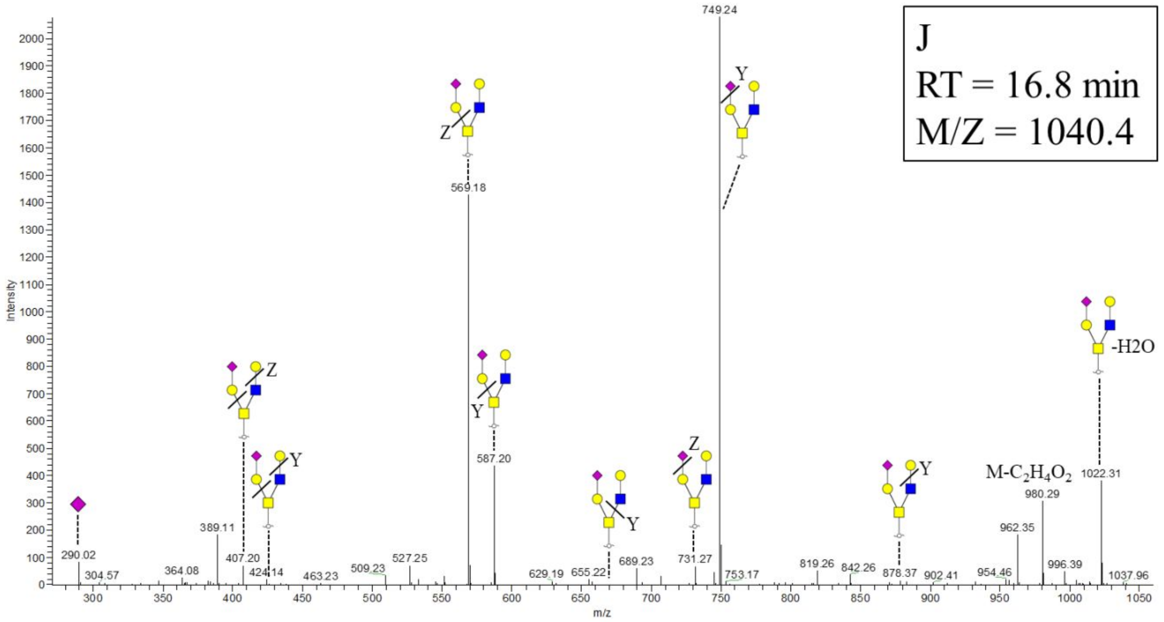
J
RT = 16.8 min
M/Z = 1040.4
Intensity
m/z
749.24
569.18
587.20
1022.31
980.29
M-C2H4O2
-H2O
Y
Z

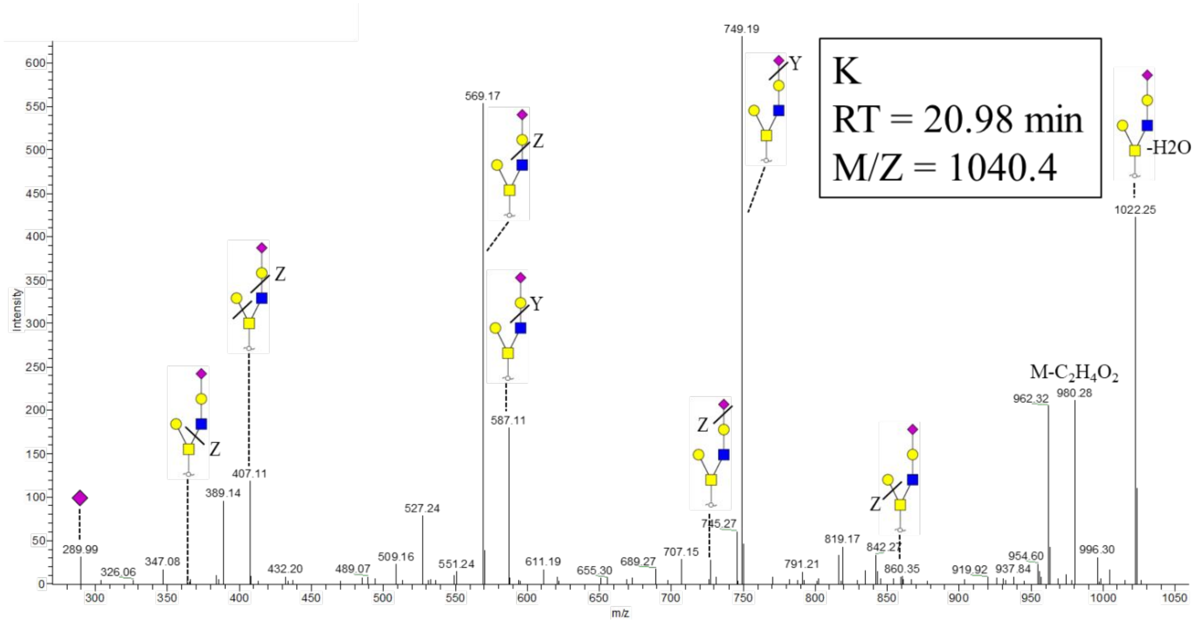
K
RT = 20.98 min
M/Z = 1040.4
Intensity
m/z
749.19
569.17
1022.25
-H2O
587.11
407.11
389.14
289.99
527.24
980.28
962.32
M-C$_2$H$_4$O$_2$
Z
Y

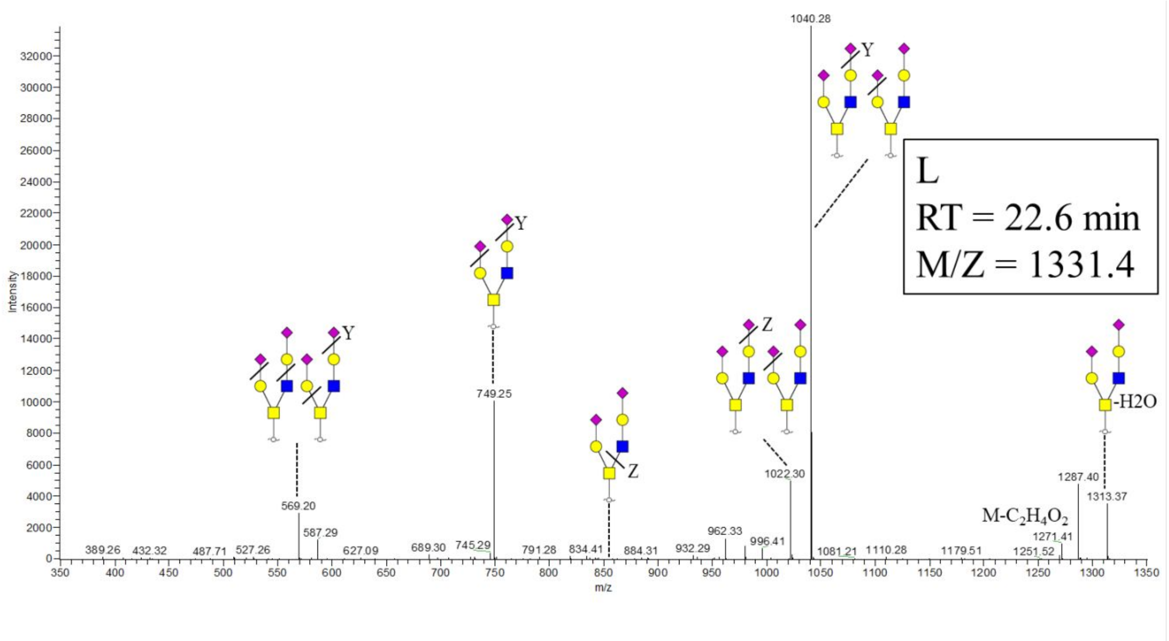
L
RT = 22.6 min
M/Z = 1331.4
Intensity
m/z
1040.28
749.25
1022.30
569.20
587.29
1313.37
1287.40
-H2O
M-C$_2$H$_4$O$_2$
Z
Y

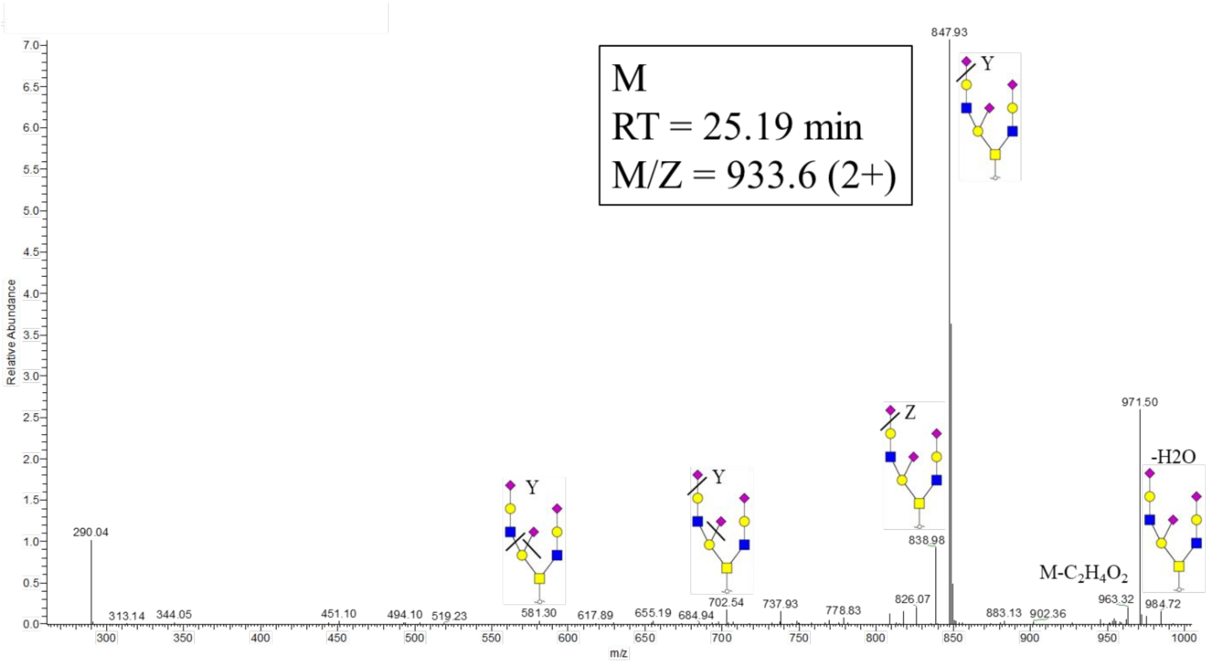
M
RT = 25.19 min
M/Z = 933.6 (2+)
Relative Abundance
m/z
847.93
971.50
-H2O
M-$C_2H_4O_2$
290.04
838.98
826.07
702.54
581.30
Y
Z

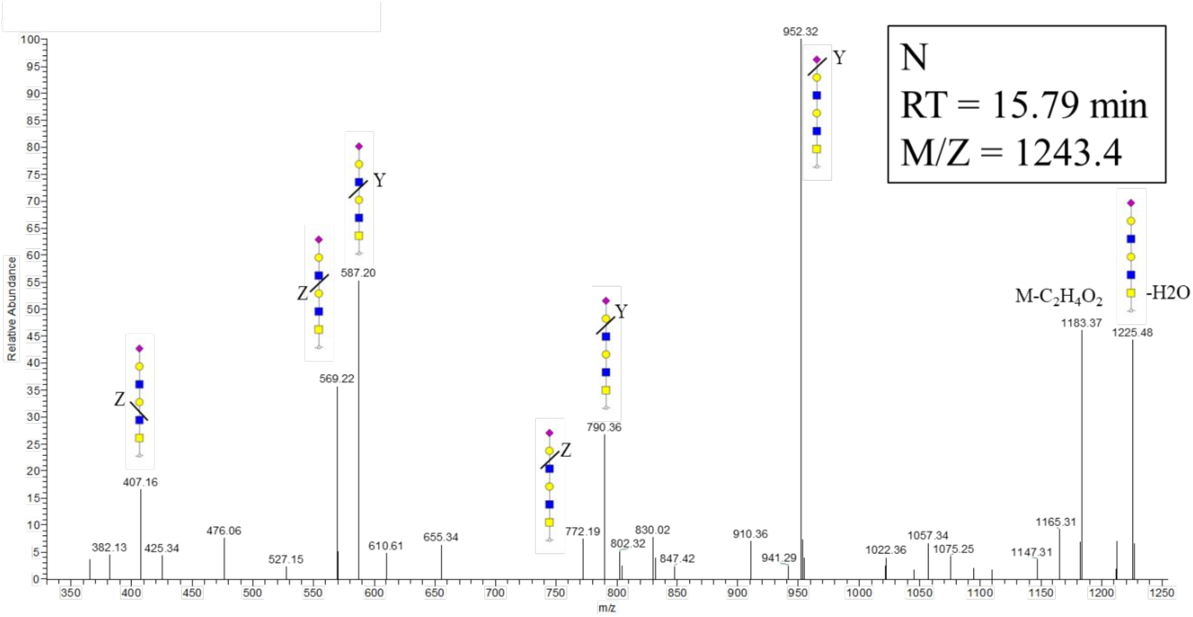
N
RT = 15.79 min
M/Z = 1243.4
Relative Abundance
m/z
952.32
587.20
569.22
790.36
407.16
1183.37
1225.48
M-$C_2H_4O_2$
-H2O
Y
Z

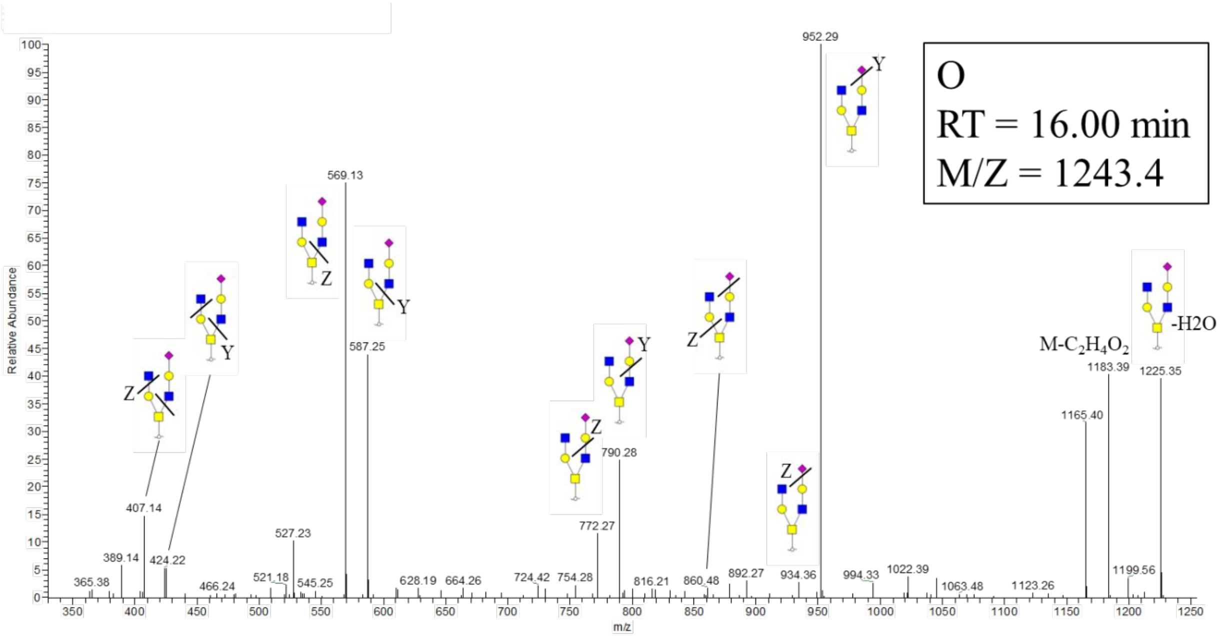
O
RT = 16.00 min
M/Z = 1243.4
Relative Abundance
m/z
952.29
569.13
587.25
407.14
424.22
389.14
365.38
466.24
527.23
521.18
545.25
628.19
664.26
724.42
754.28
772.27
790.28
816.21
860.48
892.27
934.36
994.33
1022.39
1063.48
1123.26
1165.40
1183.39
1199.56
1225.35
M-$C_2H_4O_2$
-H2O
Y
Z

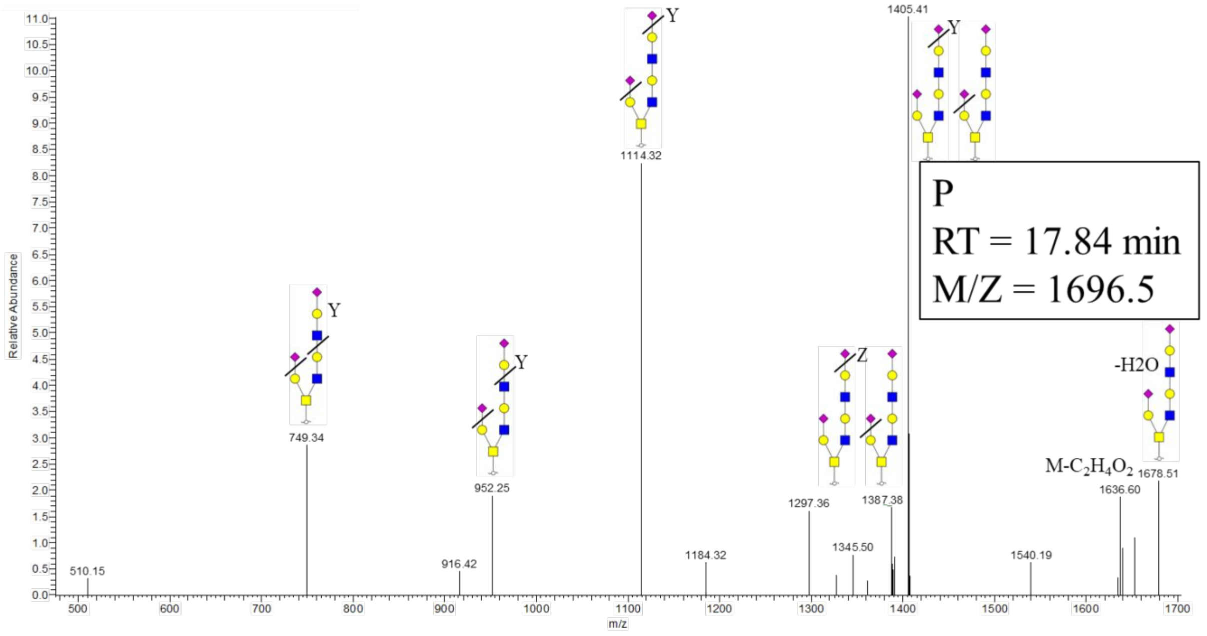
P
RT = 17.84 min
M/Z = 1696.5
Relative Abundance
m/z
1405.41
1114.32
749.34
952.25
510.15
916.42
1184.32
1297.36
1345.50
1387.38
1540.19
1636.60
1678.51
M-$C_2H_4O_2$
-H2O
Y
Z

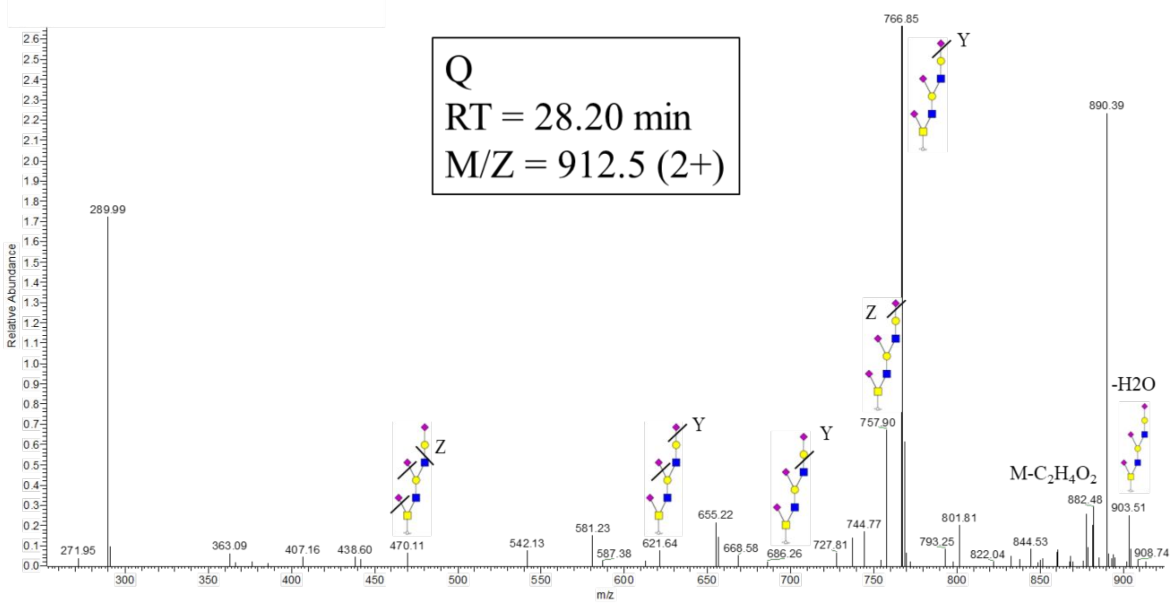
Q
RT = 28.20 min
M/Z = 912.5 (2+)
Relative Abundance
m/z
Y
Z
-H2O
M-$C_2H_4O_2$
766.85
890.39
289.99
757.90
882.48
903.51
908.74
470.11
621.64
655.22
581.23
542.13
801.81
744.77
727.81
686.26
668.58
587.38
363.09
407.16
438.60
271.95
793.25
822.04
844.53

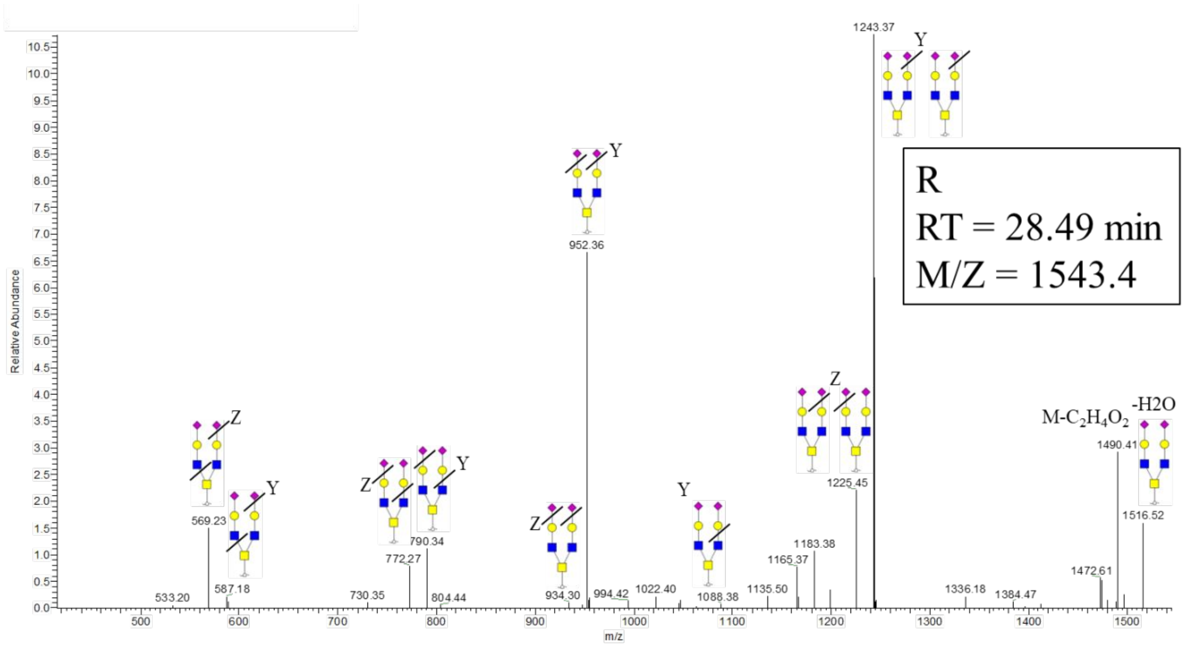
R
RT = 28.49 min
M/Z = 1543.4
Relative Abundance
m/z
Y
Z
-H2O
M-$C_2H_4O_2$
1243.37
952.36
1490.41
1516.52
1225.45
1183.38
1165.37
1472.61
569.23
587.18
772.27
790.34
934.30
1088.38
533.20
730.35
804.44
994.42
1022.40
1135.50
1336.18
1384.47

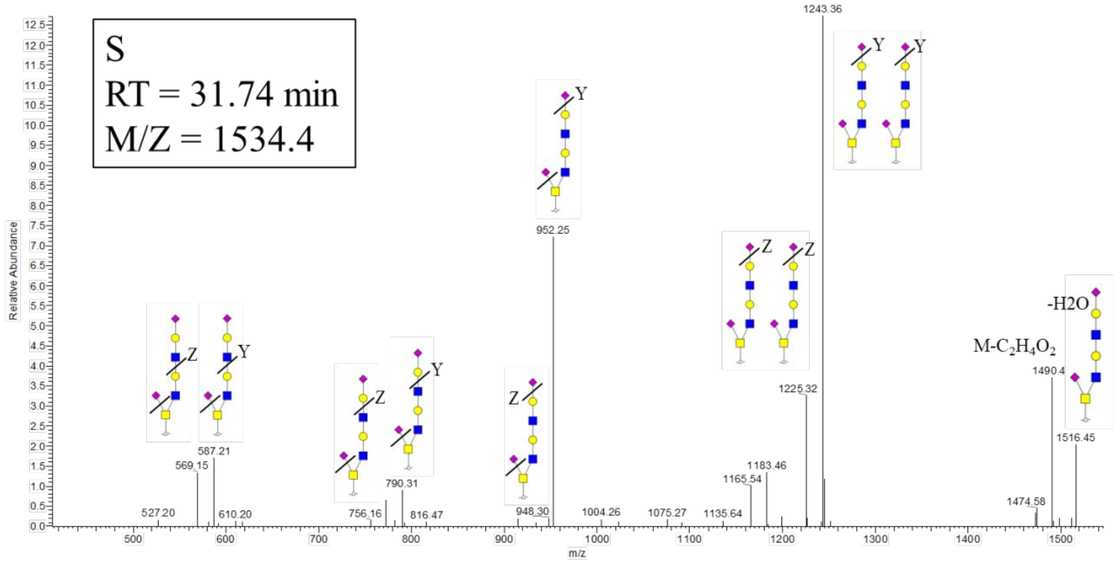

Figure S6. Example fragmentation spectra of identified O-glycans. The spectra show the different mass peaks which were used for interpretation of the glycan structures. Spectra A-J are from in vitro mucus control batch (figure 7) and spectra K-S are from in vitro mucus A. muciniphila 108 CFU/mL (figure 7). Yellow square = GalNAc, blue square = GlcNAc, blue circle = Glc, yellow circle = Gal, red triangle = Fuc, purple diamond = Neu5Ac.

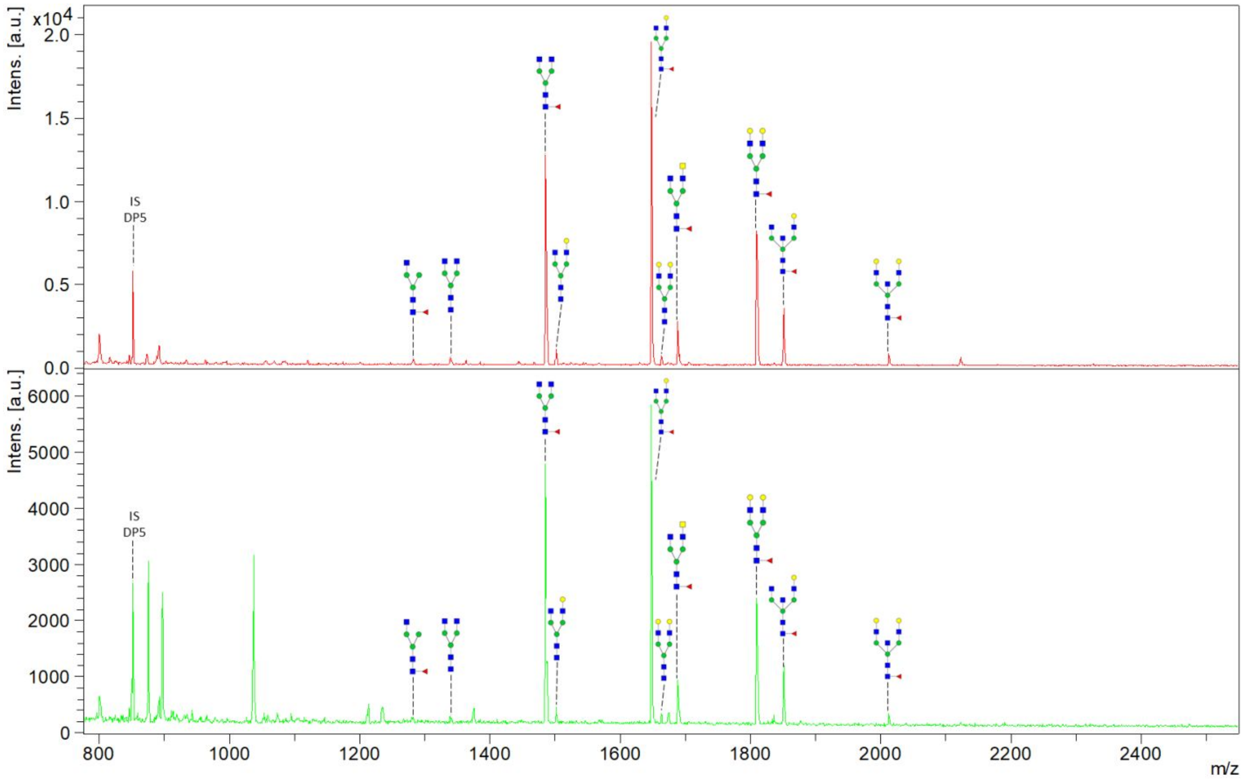

Figure S7. MALDI-TOF mass spectrum of N-glycans released from two separately prepared human IgG used as method control. Yellow square = GalNAc, blue square = GlcNAc, green circle = mannose, yellow circle = Gal, red triangle = Fuc, purple diamond = Neu5Ac.

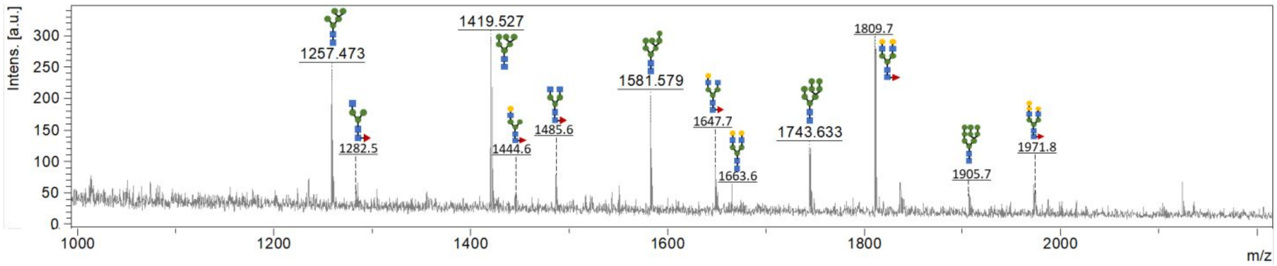

Figure S8. MALDI-TOF mass spectrum of N-glycans released from porcine stomach mucin type III used as method control. Yellow square = GalNAc, blue square = GlcNAc, green circle = mannose, yellow circle = Gal, red triangle = Fuc, purple diamond = Neu5Ac.

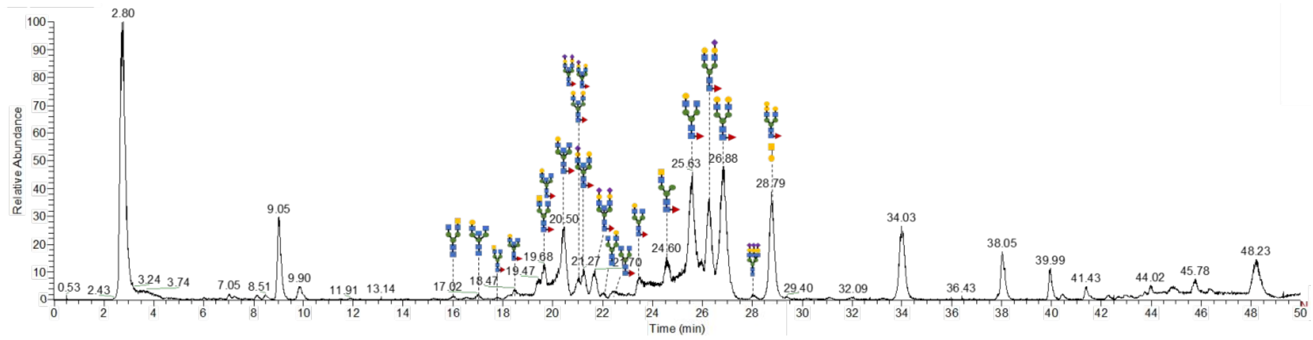

Figure S9. PGC-LC-MS/MS chromatogram of N-glycans released from human IgG used as method control. Unassigned peaks originate from the sample preparation, sample matrix, or are potential artifacts. Yellow square = GalNAc, blue square = GlcNAc, green circle = mannose, yellow circle = Gal, red triangle = Fuc, purple diamond = Neu5Ac.

Table S1. Mass spectrometric information based on MIRAGE guidelines (Kolarich et al. 2013).

Parameters	MALDI-TOF MS	PGC-LC-MS/MS
Ion source information	Bruker Autoflex MaX , Bruker MTP target plate,	Thermo Scientific HESI source, Thermo Scientific Velos Pro ion trap mass spectrometer, electrospray ionisation, negative mode
Data acquisition	*m/z* range 440 – 3000, 500 Hz laser, laser power between 40 – 45%, glycan spectra generated from sum-up 1500 satisfactory shots in 50 shot steps, hexagonal shot pattern	Two scan events: full MS scanning, negative mode, profile data, mass range 350 – 1850 *m/z* and data dependent scan, negative mode, centroid data, CID activation, 4 repeat counts, 5 s repeat duration, 50 exclusion list size, 10 s exclusion time, 1 default charge state, 2.0 *m/z* activation width, 34 eV normalised collision energy, 10 ms activation time
Ion transfer	TOF	Ion trap
Gasses	Nitrogen	Nitrogen, helium
Data acquisition and annotation	Data processing: flexAnalysis (Bruker), manual & automatic peak integration, data analysis tools: GlycoWorkbench v1.1	Data acquisition: Xcalibur (Thermo Scientific), data processing: Qual Browser (Thermo Scientific), manual peak integration, data analysis tools: GlycoWorkbench v1.1

Table S2. Sample information overview based on MIRAGE guidelines (Struwe et al 2016).

**Parameters**		**N-glycan protocol - properties**	**O-glycan protocol - properties**
Sample origin	General information	In vitro produced mucus by HT29-MTX-E12 using Semi-Wet conditions with Mechanical Stimulation in cell culture flasks	In vitro produced mucus by HT29-MTX-E12 using Semi-Wet conditions with Mechanical Stimulation in cell culture flasks
	Biologically derived	HT29-MTX-E12	HT29-MTX-E12
	Starting material	Cell culture medium including produced mucus	Cell culture medium including produced mucus
Sample processing	Isolation	Centrifuge cell culture medium, use mucus pellet, wash with H2O, PNGase-F enzymatic release	Centrifuge cell culture medium, use mucus pellet, wash with H2O, NaBH4 + NaOH reductive release
	Modification	-	-
	Purification	Solid phase extraction using porous graphitised carbon material	Solid phase extraction using C18 material and porous graphitised carbon material
	Sample definition	Released N-glycans	Released O-glycans

Table S3. Liquid chromatographic information of used set-up for the analysis of in vitro produced mucus and standards IgG and porcine stomach mucin type III based on MIRAGE guidelines (Campbell et al. 2019).

**Parameters**	**Properties**
General features	In vitro mucus produced by HT29-MTX-E12 using Semi-Wet conditions with Mechanical Stimulation in cell culture flasks. Mucins isolated and N-glycan (PNGase-F) or O-glycan (NaBH4 + NaOH) released<br>Number of replicates: 1<br>Release method:<br>O-glycan release method based on: Saldova et al. 2020, Kotsias et al. 2019<br>N-glycan release method based on: Holst et al. 2016, Jensen et al. 2016
Equipment	Thermo Scientific Vanquish ultra-high pressure liquid chromatography system<br>Thermo Scientific HESI source + Velos Pro ion trap mass spectrometer
Column details and characteristics	Thermo Scientific porous graphitised carbon Hypercarb guard column, 10 (length) x 2.1 (i.d.) mm, particle size 3 μM<br>Thermo Scientific porous graphitised carbon Hypercarb analytical column, 150 (length) x 2.1 (i.d.) mm, particle size 3 μM
Mobile phase	A: H_2O + 10 mM NH_4HCO_3<br>B: H_2O:ACN 40:60 + 10 mM NH_4HCO_3
Properties of the chromatographic run	Flow rate: 200 μL/min<br>Temperature: 40 °C<br>Injection: 10 μL<br>Gradient (50 min):<br>95% A for 3 min, 5-10% B (3-15 min), 0-0 % B (15-25 min), 20-25% B (25-30 min), 25-40% B (30-37 min), 40-95% B (37-37.5 min), 95% B for 4.5 min, 95-5% B (42-42.5 min), 95% A for 7.5 min
Data acquisition and annotation	Instrument controller: Xcalibur (Thermo Scientific)<br>Data acquisition: Xcalibur (Thermo Scientific)<br>Data processing: Qual Browser (Thermo Scientific), manual peak integration<br>Data analysis tools: GlycoWorkbench v1.1

Table S4. Peak area and calculated relative abundance percentage based on peak area of identified O-glycans released from *in vitro* produced mucus obtained from HT29-MTX-E12 cells grown under Semi-Wet conditions with Mechanical Stimulation exposed to different concentrations of pasteurised *A. muciniphila*. Yellow square = GalNAc, blue square = GlcNAc, yellow circle = Gal, blue circle = Glc, red triangle = Fuc, Purple diamond = Neu5Ac

Control				A. muciniphila 10x				A. muciniphila 100x				A. muciniphila 1000x			
	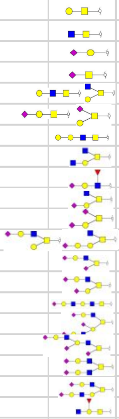	Peak area	Rel % peak area		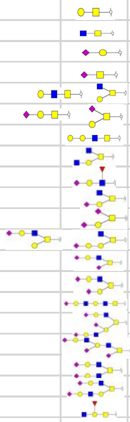	Peak area	Rel % peak area		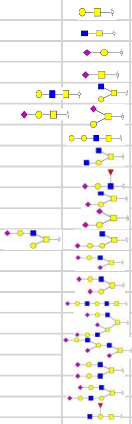	Peak area	Rel % peak area		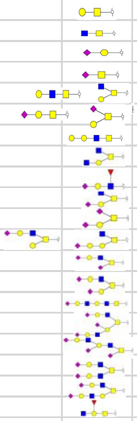	Peak area	Rel % peak area
		883615.5	5.2			1991094.5	4.1			1421664.9	8.2			1828461.2	11.9
		129541.0	0.8			426461.4	0.9			275269.6	1.6			346582.9	2.3
		1250818.7	7.4			2775602.8	5.7			1113260.7	6.5			881443.3	5.8
		300010.3	1.8			658030.1	1.3			265408.2	1.5			214969.6	1.4
		167237.3	1.0			565303.7	1.2			224082.2	1.3			229286.6	1.5
		9964194.0	58.9			28049728.7	57.2			9398186.9	54.5			7652066.5	50.0
		96660.7	0.7			330523.0	0.9			204113.0	1.4			269767.1	2.1
		32389.3	0.2			151791.5	0.3			40235.4	0.2			107633.5	0.7
		118617.3	0.7			645928.9	1.3			295459.8	1.7			554850.2	3.6
		350147.7	2.1			1239951.7	2.5			349845.7	2.0			217640.2	1.4
		2223480.5	13.2			7008808.5	14.3			2216309.1	12.9			1678206.8	11.0
		111836.7	0.7			471378.8	1.0			122398.0	0.7			107573.2	0.7
		54555.3	0.3			143121.3	0.3			53846.4	0.3			179682.6	1.2
		1090402.3	6.4			3551970.1	7.2			1053875.6	6.1			836309.0	5.5
		94549.3	0.6			134094.4	0.3			47907.6	0.3			40746.4	0.3
		0.0	0.0			218802.7	0.4			52395.9	0.3			46014.9	0.3
		0.0	0.0			52475.5	0.1			0.0	0.0			0.0	0.0
		0.0	0.0			188331.2	0.4			0.0	0.0			0.0	0.0
		0.0	0.0			291715.8	0.6			52167.7	0.3			44925.5	0.3
		18505.0	0.1			52414.0	0.1			28687.0	0.2			28112.0	0.2

Table S5. Peak area and calculated relative abundance percentage based on peak area of identified O-glycans released from *in vitro* produced mucus obtained from HT29-MTX-E12 cells grown under Semi-Wet conditions with Mechanical Stimulation exposed to different pasteurised bacteria *B. fragilis*, *R. gnavus*, and *A. muciniphila* at concentration of 10E6 CFU/mL. Yellow square = GalNAc, blue square = GlcNAc, yellow circle = Gal, blue circle = Glc, red triangle = Fuc, Purple diamond = Neu5Ac

Control				B. fragilis 10x				R. gnavus 10x				A. muciniphila 10x			
		Peak area	Rel % peak area			Area	Rel % peak area			Area	Rel % peak area			Peak area	Rel % peak area
		883615.5	5.2			594732.0	3.3			1025308.5	2.4			1991094.5	4.1
		129541.0	0.8			114031.9	0.6			204698.0	0.5			426461.4	0.9
		1250818.7	7.4			1212680.0	6.7			2302215.5	5.5			2775602.8	5.7
		300010.3	1.8			288458.2	1.6			580574.0	1.4			658030.1	1.3
		167237.3	1.0			226178.7	1.3			206789.9	0.5			565303.7	1.2
		9964194.0	58.9			10483551.1	58.1			24884141.9	59.3			28049728.7	57.2
		96660.7	0.7			90353.3	0.7			149926.9	0.4			330523.0	0.9
		32389.3	0.2			201510.3	1.1			364924.2	0.9			151791.5	0.3
		118617.3	0.7			336351.1	1.9			996103.4	2.4			645928.9	1.3
		350147.7	2.1			2733277.7	15.1			6365487.2	15.2			1239951.7	2.5
		2223480.5	13.2			141768.6	0.8			530440.6	1.3			7008808.5	14.3
		111836.7	0.7			65985.3	0.4			169759.8	0.4			471378.8	1.0
		54555.3	0.3			1195214.5	6.6			3192557.7	7.6			143121.3	0.3
		1090402.3	6.4			80795.4	0.4			134910.7	0.3			3551970.1	7.2
		94549.3	0.6			44558.0	0.2			77621.6	0.2			134094.4	0.3
		0.0	0.0			56672.6	0.3			187002.8	0.4			218802.7	0.4
		0.0	0.0			25814.0	0.1			85412.6	0.2			52475.5	0.1
		0.0	0.0			60437.0	0.3			122454.1	0.3			188331.2	0.4
		0.0	0.0			59476.9	0.3			293719.4	0.7			291715.8	0.6
		18505.0	0.1			15629.0	0.1			28132.0	0.1			52414.0	0.1

Table S6. Peak area and calculated relative abundance percentage based on peak area of identified N-glycans released from *in vitro* produced mucus obtained from HT29-MTX-E12 cells grown under Semi-Wet conditions with Mechanical Stimulation exposed to different pasteurised bacteria *B. fragilis*, *R. gnavus*, and *A. muciniphila* at concentration of 10E6 CFU/mL. Yellow square = GalNAc, blue square = GlcNAc, yellow circle = Gal, blue circle = Glc, red triangle = Fuc, Purple diamond = Neu5Ac

		Intensity					Relative abundance based on intensity			
		Control	*B. fragilis* 10E6 CFU/mL	*R. gnavus* 10E6 CFU/mL	*A. muciniphila* 10E6 CFU/mL		Control	*B. fragilis* 10E6 CFU/mL	*R. gnavus* 10E6 CFU/mL	*A. muciniphila* 10E6 CFU/mL
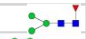	1079	995	181	109	138		4.6	4.3	6.9	6.8
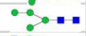	1095	370	207	55	93		1.7	4.9	3.5	4.6
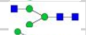	1136	461	116	59	66		2.1	2.7	3.8	3.3
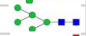	1257	5056	870	283	456		23.3	20.4	18.0	22.5
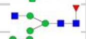	1282	785	220	74	85		3.6	5.2	4.7	4.2
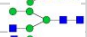	1298	137	56	42	51		0.6	1.3	2.7	2.5
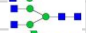	1339	463	141	65	76		2.1	3.3	4.1	3.8
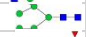	1419	2478	431	115	172		11.4	10.1	7.3	8.5
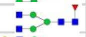	1485	1777	316	108	151		8.2	7.4	6.9	7.5
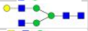	1501	90	55	27	35		0.4	1.3	1.7	1.7
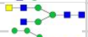	1542	303	123	48	71		1.4	2.9	3.1	3.5
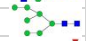	1581	1803	275	95	104		8.3	6.5	6.0	5.1
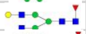	1647	125	56	30	30		0.6	1.3	1.9	1.5
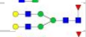	1663	75	75	30	56		0.3	1.8	1.9	2.8
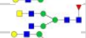	1688	2712	358	93	110		12.5	8.4	5.9	5.4
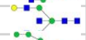	1704	53	51	45	40		0.2	1.2	2.9	2.0
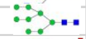	1743	2397	357	100	99.4		11.0	8.4	6.4	4.9
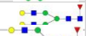	1809	113	37	27	40		0.5	0.9	1.7	2.0
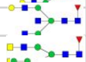	1850	88	46	29	29		0.4	1.1	1.8	1.4
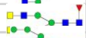	1891	817	92	49	45		3.8	2.2	3.1	2.2
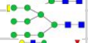	1905	518	119	54	44		2.4	2.8	3.4	2.2
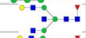	1996	69	41	0	32		0.3	1.0	0.0	1.6
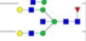	2012	39	34	36	0		0.2	0.8	2.3	0.0
	SUM	21724	4257	1573	2023.4		100.0	100.0	100.0	100.0
